# Current Status and Future Directions of Botulinum Neurotoxins for Targeting Pain Processing

**DOI:** 10.3390/toxins7114519

**Published:** 2015-11-04

**Authors:** Sabine Pellett, Tony L. Yaksh, Roshni Ramachandran

**Affiliations:** 1 Department of Bacteriology, University of Wisconsin, 6340 Microbial Sciences Building, 1550 Linden Dr., Madison, WI 53706, USA; E-Mail: sabine.pellett@wisc.edu; 2 Department of Anesthesiology 0818, University of California, 214 Dickinson St., San Diego, CA 92103, USA; E-Mail: tyaksh@ucsd.edu

**Keywords:** pain, botulinum neurotoxin, BoNT, spinal cord, primary afferent, glia, neurotransmitter, SNAREs

## Abstract

Current evidence suggests that botulinum neurotoxins (BoNTs) A1 and B1, given locally into peripheral tissues such as skin, muscles, and joints, alter nociceptive processing otherwise initiated by inflammation or nerve injury in animal models and humans. Recent data indicate that such locally delivered BoNTs exert not only local action on sensory afferent terminals but undergo transport to central afferent cell bodies (dorsal root ganglia) and spinal dorsal horn terminals, where they cleave SNAREs and block transmitter release. Increasing evidence supports the possibility of a trans-synaptic movement to alter postsynaptic function in neuronal and possibly non-neuronal (glial) cells. The vast majority of these studies have been conducted on BoNT/A1 and BoNT/B1, the only two pharmaceutically developed variants. However, now over 40 different subtypes of botulinum neurotoxins (BoNTs) have been identified. By combining our existing and rapidly growing understanding of BoNT/A1 and /B1 in altering nociceptive processing with explorations of the specific characteristics of the various toxins from this family, we may be able to discover or design novel, effective, and long-lasting pain therapeutics. This review will focus on our current understanding of the molecular mechanisms whereby BoNTs alter pain processing, and future directions in the development of these agents as pain therapeutics.

## 1. Introduction

Botulinum Neurotoxins (BoNTs) are the most potent toxins known to humankind and are the causative agent of the serious and potentially fatal paralytic disease botulism [1]. About 35–40 years ago, it was shown that local intramuscular injection of low doses of BoNTs resulted in local, long-lasting, but reversible paralysis of the injected muscle [2,3]. This property of BoNTs proved useful for treatment of strabismus [3,4]. These landmark studies together with the development of purification techniques and standardized detection assays [5] led to the development of BoNTs as widely used pharmaceuticals to produce paralysis of additional therapeutic targets, including the facial musculature for cosmetics and large muscle groups to manage debilitating muscle spasms (as in torticollis and back spasms) [6,7]. Other applications that have evolved and for which FDA approval has been granted include management of hyperactive bladder and hyperhidrosis [6,7]. Common to all of these applications is that the BoNTs are taken up into the motor neuron terminal at the neuromuscular junction or parasympathetic axon terminal, where the toxin acts to block the release of acetylcholine [8]. Importantly, these effects are consistently achieved by a localized action after local delivery without systemic redistribution [2,3,4]. Early on in the use of BoNTs for treatment of spasticity disorders it was incidentally observed that the pain associated with muscle spasms was significantly attenuated to a degree that exceeded that which would have been anticipated from the simple reduction of muscle contracture [9]. Of particular interest was the appreciation several years later that pericranial injection of BoNT/A1 and B1 could relieve symptoms in some forms of chronic migraine [10,11,12,13,14,15,16,17,18,19,20,21,22]; however, some contradictory findings were also reported [23,24,25]. Intramuscular BoNT/A1 injections across the head and neck was FDA-approved as a treatment for chronic migraine headaches in 2010. A robust literature now shows that the local delivery of BoNT/A1 or BoNT/B1 has profound effects upon a variety of pain states in preclinical models, and, importantly, in clinical pain syndromes. In this review, we will consider i) the mechanisms of action of BoNT/A1 and /B1 in altering cellular function, ii) the effects of BoNT/A1 and /B1 on pain behavior phenotypes in preclinical models and in human pain states, iii) the likely mechanisms underlying this action and iv) future directions for the development of novel BoNT-based therapeutics to target nociceptive processing, and the value of investigating the diversity of the large family of BoNTs in this endeavor. 

## 2. Botulinum Neurotoxin Structure and Function

**Figure 1:**
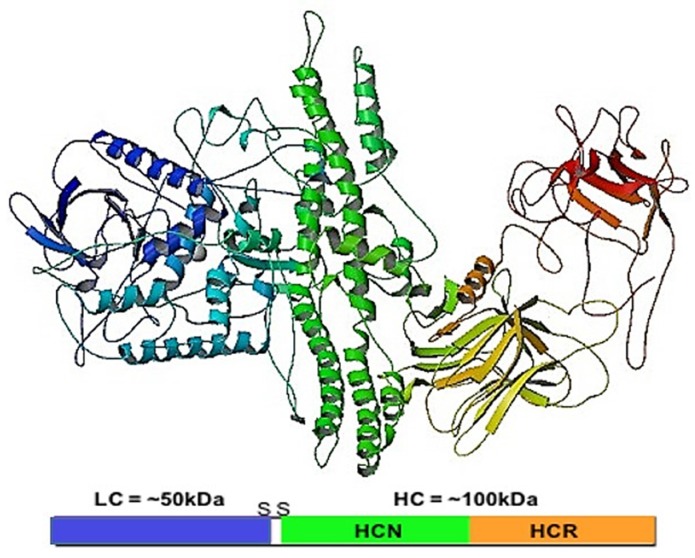
Botulinum Neurotoxin A1 (BoNT/A1): The 50 kDa light chain (LC) (blue) is linked to the 100 kDa heavy chain (HC) (green, yellow, and red). The HC is functionally divided into the translocation domain (HCN) (green) required for transport of the LC from the endosome into the cell cytosol, and the receptor binding domain (HCR) (yellow and red) through which BoNT binds to the cell surface. Crystal structure image from the Protein databank doi:10.2210/pdb3bta/pdb [36].

**Figure 2:**
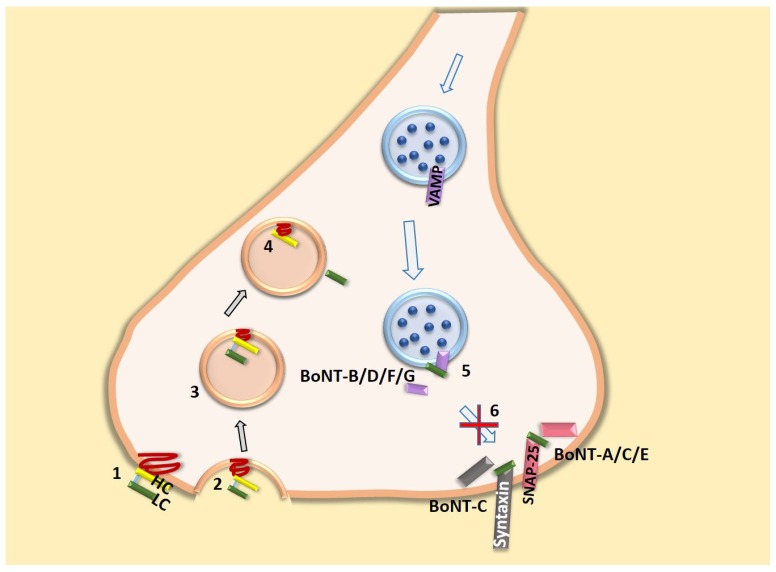
BoNT uptake mechanism and targets: BoNT consists of heavy chain (HC) and light chain (LC). Heavy chain binds to the ganglioside and protein receptors (1) and is endocytosed (2,3). The acidic environment in the endosomes leads to conformational changes resulting in membrane insertion of the HC and LC translocation into the cell cytosol (4). In the cytosol, the disulfide bond linking HC and LC is reduced, releasing the LC into the cytosol. The LC of BoNT-B/D/F/G specifically cleaves VAMP on the vesicle, BoNT-A/C/E cleaves SNAP-25 and BoNT-C also cleaves syntaxin on plasma membranes (5), thus inhibiting the vesicular fusion and blocking the neurotransmitter release (6). Not shown is the potential role of cytosolic SNAREs mediating the trafficking of receptor and channel subunit protein to the membrane in lipid raft scaffolding.

BoNTs are a large family of proteins that are produced by a diverse group of gram positive, spore forming anaerobic bacteria termed *Clostridium botulinum* and by a few strains of *Clostridium butyricum*, *sporogenes*, and *baratii* [26,27]. At least 40 different subtypes of BoNTs have been described today. The current nomenclature differentiates newly identified BoNTs as novel subtypes based purely on an amino acid difference greater than 2.5% [28], and many more variants exist that are not classified as unique subtypes. The BoNTs are classified into seven serotypes (A though G) based primarily on antigenic specificity [29], and the subtypes within each serotype are denoted by a number following the letter. All BoNTs are modular proteins constructed of a 100 kDa heavy chain (HC) and a 50 kDa light chain (LC) linked by a disulfide bond (Figure 1). The HC is divided into a C-terminal receptor binding domain (HCR) and an N-terminal translocation domain (HCN) [30]. Cell entry by BoNTs proceeds via a multi-step process (Figure 2). The HCR of BoNTs binds to specific protein and ganglioside receptors on the cell surface, leading to endocytosis of the HC-LC complex. In the acidic environment of the endocytic vesicle, protonation causes a conformational shift in the BoNT protein resulting in incorporation of the HC into the endocytic vesicle membrane to form a channel through which the LC is translocated into the cytosol [30,31,32]. The disulfide bond is reduced inside the cell’s cytosol, releasing the LC to refold to an enzymatically active conformation [8,33]. The BoNT LC is a zinc-dependent endoprotease that, once inside the cell, cleaves intracellular SNAREs (soluble N-ethylmaleimide -sensitive-factor attachment protein receptors) at highly specific consensus sites. This cleavage prevents SNARE-mediated protein transport and transmitter release [1,34], which, at the neuromuscular junction, results in a failure of muscle innervation and thus flaccid paralysis. An important characteristic of BoNT LC is that it is capable of persisting in an active configuration in the cytosol for days to months, depending on the BoNT serotype. During that time, the LC continues to cleave its target SNARE, which accounts for the associated duration of action of the respective toxin [8,35].

## 3. Peripheral Delivery of Botulinum Toxins and Nociception in Preclinical and Human Models

Pain has a profound effect upon the well-being of the human and animal, and treatment of pain remains a high priority. Here, we will consider the actions of BoNTs delivered peripherally into the skin and muscles to locally alter pain states. In this discussion, it is heuristically useful to consider the effects of these toxins on the distinct nociceptive processing generated by acute high intensity stimulation, tissue injury/inflammation and nerve injury. Mechanistic studies with clinical implication involving the intrathecal delivery of these toxins will also be considered in the targeting of these toxins. We note that the majority of work to date has focused on the commercially available BoNT/A1 and /B1 serotypes. While these enabling observations are exciting, we argue that they make the case for further investigations into the actions on pain processing of other serotypes and subtypes. Table 1 and Table 2 summarize the clinical and pre-clinical findings of BoNT in different pain models.

**Table 1:** Summarizes the clinical studies examining the effectiveness of botulinum neurotoxins (BoNT)/A1/B1 in several pain conditions.

No	Type of Pain Condition/Model	BoNT Serotype	Outcome	Interpretations	References
1)	**Migraine**				
	Acute Migraine (<15 attacks/month)	BoNT/A1	Positive	Reductions in migraine severity and headache frequency	[15,16,17]
			Negative	No significant differences observed between the placebo and BoNT treatment group. Few studies observed a trend however not significant	[23,24]
	Chronic Migraine (>15 attacks/month)	BoNT/A1	Positive	Pooled result of PREEMPT trials favored both primary and secondary endpoints. Reduction in cephalic allodynia associated with chronic migraine	[13,14,18,21,22]
			Negative	Mild or no effect was observed in this randomized controlled study	[25]
		BoNT/B1	Positive	Chronic migraineurs significantly responded to Rimabotulinumtoxin (BoNT/B1)	[19,20]
2)	Chronic joint pain	BoNT/A1	Positive	BoNT/A1 showed significant effect in treating refractive shoulder joint pain	[37,38]
			Positive	Efficacy in painful knee and joint arthritis	[39,40]
3)	**Neuropathic pain**				
	Mononeuropathy	BoNT/A1	Positive	BoNT treatment was effective in treating trigeminal neuralgia and peripheral nerve injury	[41,42,43,44,45,46,47,48]
	Polyneuropathy	BoNT/A1	Positive	Effective in cases of post-herpetic neuralgia Efficacy reported for diabetic neuropathy	[49,50,51,52]
4)	Chronic low back pain	BoNT/A1	Positive	Treatment in paraspinal muscles reduced pain in refractory low back pain patients	[53,54]
5)	Myofascial pain	BoNT/A1	Positive	BoNT reduced focal myofascial pain syndrome	[55,56]
6)	**Evoked nociception model**				
	Capsaicin	BoNT/A1	Positive	BoNT inhibited capsaicin induced flare alone or both flare and evoked nociception	[57,58,59,60]
			Negative	Showed no effect on any end points	[61,62,63]
	Glutamate	BoNT/A1	Positive	Reduced glutamate evoked pain and local increase in skin blood flow	[64]
	Thermal Injury	BoNT/A1	Negative	Evoked primary or secondary hyperalgesia was not altered by BoNT	[65]
7)	Acute thresholds	BoNT/A1	Negative	No effect upon normal thermal and mechanical pain thresholds in quantitative testing paradigm	[57,61,62,63]

**Table 2:** Pre-clinical (*in vivo* and *in vitro*) studies examining effectiveness of BoNT/A1 and B1 in several pain models.

No	Type of Pain Condition/Model	BoNT Serotype	Species	Outcome	Interpretations	References
1)	**Inflammatory pain models**					
	Formalin	BoNT/A1/B1	Rat/mouse	Positive	Peripheral BoNT shows little or no effect on phase I with long term inhibition in phase II formalin flinching and neuronal c-fos activation. Inhibition of neurotransmitter release. Cleavage of SNARES in DRG/TG and spinal cord	[66,67,68,69,70,71,72,73]
	Capsaicin	BoNT/A1/B1	Rat/mouse	Positive	Peripheral BoNT reduces capsaicin evoked flare, nociceptive behavior and inhibition of neurotransmitter release. Cleavage of SNARES in TG and nucleus caudalis	[67,74,75]
	Carrageenan	BoNT/A1	Rat	Negative	Lack of effect on peripheral inflammation and pain	[76,77]
		BoNT/A1/A2	Rat	Negative	No effect of BoNT was observed in carrageenan evoked flare and plasma extravasation	[77,78,79]
	Arthritis	BoNT/A1	Rat	Positive	BoNT reduced allodynia in CFA induced knee arthritic animals	[80]
		BoNT/A1	Dogs	Positive	Intraarticular BoNT reduced indices of pain	[81,82,83]
2)	**Neuropathic pain**					
	Mononeuropathy (peripheral and infraorbital nerve ligation, ventral root transection)	BoNT/A1/B1	Rat/mouse	Positive	BoNT reduced allodynia following peripheral treatment. Suggests central action of BoNT	[74,84,85,86,87]
	Polyneuropathy (Chemotherapeutics, diabetes)	BoNT/A1	Rat/mouse	Positive	Reduced thermal and mechanical hyperalgesia with bilateral effects	[78,84,88,89]
3)	Trigeminal pain models	BoNT/A1/B1	Rat/mouse	Positive	Orofacial BoNT reduced capsaicin evoked nocifensive behavior, inhibited trafficking of TRPV1 to plasma membrane, cleavage of SNARES	[75,90]
					Decreased mechanical sensitivity of temporal muscle nociceptors. Inhibited responses to mechanical stimulation of dura to supra threshold forces	[91]
	***In vitro* (cell/organ culture studies)**					
1)	Dorsal root ganglion sensory neuron cell culture	BoNT/A1	Rat	Positive	BoNT cleaves neuronal SNARES and prevents release of neurotransmitters	[92,93]
2)	Trigeminal sensory neuron organ/cell culture	BoNT/A1	Rat	Positive	BoNT inhibits CGRP secretion from TG neuronal culture	[94]
					Modify expression of inflammatory markers both in neurons and glial cells	[95]
3)	Trigeminal satellite glial cell culture	BoNT/A1	Rat	Positive	BoNT cleaves glial SNARES and inhibited glutamate release	[96]

### 3.1. Acute Nociception 

#### 3.1.1. Mechanisms

Acute high intensity stimuli lead to a pain state referred to the site of stimulation. This pain state reflects the intensity-dependent activation of populations of small primary afferents (A∂ and C fibers) by high intensity thermal or mechanical stimuli, which release excitatory transmitters (amino acids and peptides) from their terminals in the spinal dorsal horn. These transmitters activate second order spinal dorsal horn neurons that project supraspinally [97]. Absent the stimulus, the afferent traffic resolves and the pain sensation abates.

#### 3.1.2. Botulinum Toxins

Intraplantar delivery of both BoNTs, A1 and B1, at doses that do not obstruct motor function, have no effect upon the behavioral escape response otherwise evoked by acute high intensity thermal or mechanical stimuli (pin prick), or chemical stimulation such as evoked paw withdrawal or local chemical irritants (e.g., phase 1 of the biphasic flinching response otherwise evoked by intraplantar or orofacial formalin) [66,67,68,69,70,71,72,73]. It is important to emphasize that nociceptive threshold testing must occur in the absence of disabling motor function. Such end points would include absence of changes driven by non-noxious stimulation, including corneal touch-evoked blink, hind paw placing and stepping reflexes, weight bearing or symmetrical ambulation. Adhering to such robust end points would indicate that any observed changes in escape latency or pain thresholds were not the result of a failure in the motor-dependent ability to produce the analgesic end point (e.g., paw withdrawal/flinching) [66,67,71,98]. It should be noted that in general the doses employed in these studies are typically in the range of 0.5–1 U, where 1 U is the dose that leads to mortality in half of the mice upon intraperitoneal injection. This reflects upon the fact that the intraplantar or subcutaneous injections result in a restricted redistribution as compared to that achieved with intraperitoneal delivery [67,84,99].

In human studies, subcutaneous BoNT/A1, as in the preclinical studies, had no effect upon normal thermal or mechanical pain thresholds in quantitative sensory testing paradigms [57,61,62,63]. As will be reviewed further below, given the effects of BoNT/A1 and BoNT/B1 on afferent transmitter release, these observations, showing no change in acute thresholds, are unexpected. 

### 3.2. Tissue Injury/Inflammation

#### 3.2.1. Mechanisms

In the face of tissue injury and inflammation, the associated pain state is characterized by: i) an ongoing sensation that persists in the absence of the injuring stimulus; and, ii) the presence of a hyperalgesia where an innocuous or mildly noxious stimulus is perceived to be aversive or noxious (e.g., hyperalgesia). Typically, once the underlying pathology is resolved (e.g., wound healing, resolution of inflammation), the pain and hyperalgesia may also resolve. The ongoing pain state and enhanced responsiveness reflects two elements. First, nociceptive afferents innervating the damaged/inflamed tissue are acted upon by active factors released by vascular cells (neutrophils, lymphocytes, platelets) and resident cells of the innate immune response (macrophages and mast cells) [100]. These factors act upon eponymous receptors expressed by many small afferents to depolarize the terminals leading to continuous afferent input and an enhanced response of the terminal to subsequent afferent stimulation [101]. This terminal sensitization is secondary to the activation of terminal kinases which phosphorylate terminal receptors (such as for the bradykinin receptor) [102] and ion channels (e.g., TRPV1, voltage gated sodium and calcium channels) [103,104,105,106,107]. Secondly, spinal systems in the face of ongoing high frequency small afferent activation can display an enhanced input-output function, referred to as wind up and/or central sensitization [101,108]. The pharmacology of this central facilitation is equally complex and reflects: i) a progressive depolarization of the second order neuron; ii) activation (phosphorylation) of spinal kinases that enhance the reactivity of the second order membrane by phosphorylating membrane channels and receptors [109,110,111]; iii) changes in the transport of various excitatory receptor and channel subunits to the membrane [112,113,114], and iv) activation of dorsal horn non-neuronal (astrocyte and microglia) cells leading to release of a variety of pro excitatory products [115,116]. These cascades are ubiquitous and relevant to all inflammatory states, including those arising from the skin, muscle, bone, and visceral tissues such as the gut and the meninges. These pain states have several important characteristics. First, pain states arising from viscera (e.g., irritable bowel) and bone (e.g., cancer), for example, display a referred pain component, in which the visceral/bone afferent input is referred to the superficial (cutaneous) dermatome which projects by a somatic afferent to the same spinal second order neurons. This referral model is particularly relevant to phenomena such as migraine, wherein meningeal afferents activated by local meningeal inflammatory processes activate neurons in the nucleus caudalis that also receive input from cutaneous cranial afferents [117]. Second, it has become appreciated that, in the face of persistent inflammation, the inflammatory pain phenotype may progress to one that involves nerve injury. In the case of joint inflammation, pain may continue after resolution of the inflammation. The mechanism of this transition to a chronic pain state is not well understood, but several changes are relevant. Persistent peripheral inflammation may lead to: i) increased expression of ATF-3 in the dorsal root ganglion (indicative of an injury response); ii) spinal cord glial activation [118,119]; and, iii) peripheral terminals may begin to express nerve injury epitopes suggestive of sprouting [120]. Thus, mechanisms of pain associated with tissue and nerve injury may occur in conditions such as osteoarthritis where inflammatory markers (joint volume, neutrophils) may be minimally present [121,122,123]. 

#### 3.2.2. Botulinum Toxin 

Unilateral intraplantar delivery of BoNT/A1 and /B1 at doses of 0.5 U or less exhibit a local, typically homolateral anti-hyperalgesia as measured by thermal and mechanical thresholds in rodent models of inflammation (e.g., intraplantar carrageenan, or formalin) [66,67,76,78]. These effects, where reported, have an onset of 2 to 24 hours and a duration of action in excess of 14 to 21 days [67]. In horses, intra-articular BoNT/A1 can attenuate lameness in a model of acute synovitis [124]. Similarly, in dogs with chronic osteoarthritis, intra-articular BoNT/A1 was found to reduce indices of pain [81,82,83]. The typical ipsilateral effect emphasizes that the BoNTs did not exert a bilateral effect and thus were not likely due to a simple systemic redistribution at the employed doses. We note that, while ipsilaterally delimited effects have been observed, particularly at lower doses, bilateral anti-hyperalgesia [78,88,89] and bilateral muscle relaxation [125] after unilateral delivery into the paw have been reported. The likelihood of multi-segmental effects on motor and sensory function in humans has been expertly reviewed and discussed in detail elsewhere [126]. 

In humans, myofascial pain syndromes, characterized by activation of multiple bilateral local pain state trigger points, have been shown to be attenuated by local delivery of BoNT/A1 [127,128]. However, systematic trials appear to be lacking [129,130]. Intra-articular delivery of BoNT/A1 has been shown to have attenuating effects upon inflammatory and chronic joint pain in the shoulder [37,38,39,40,131]. Paraspinal muscular application of BoNT/A1 reduced refractory low back pain in the majority of patients [53].

To further characterize the effects of peripheral BoNTs on human pain behavior, validated experimental models have been examined. Subcutaneous capsaicin, resulting in persistent small afferent activation, evokes a local pain sensation referred to the site of injection, a primary and secondary hyperalgesia and an increase in local cutaneous blood flow (flare). Pretreatment with subcutaneous BoNT/A1 was reported to diminish pain (hyperalgesia) and blood flow (flare) [57]. Others reported either an effect only upon capsaicin induced flare [132] or no effect on any end point [61,62,63]. Studies examining the effects of pretreatment with subcutaneous BoNT/A1 for the pain and local increase in skin blood flow evoked by subcutaneous glutamate observed a significant inhibition with peak effects by seven days and a return to baseline by 60 days [64]. Following focal thermal injury, the associated primary and secondary hyperalgesia was not altered by BoNT/A1 in a double-blinded crossover study [65]. Taken together, these data reveal mixed indications of efficacy of BoNTA1 in reducing inflammatory pain states, and further studies are needed.

### 3.3. Peripheral Nerve Injury

#### 3.3.1. Mechanisms 

Injury to the peripheral nerve trunk or terminals (e.g., yielding mono and poly neuropathies, respectively) leads to a paradoxical pain state in which the individual reports an ongoing pain condition (dysesthesia) and an exaggerated response to light touch ipsilateral to the nerve injury or bilateral. The tactile hypersensitivity is mediated by the activation of large (low threshold) Aβ axons. In animal models, mononeuropathies arise from ligation of nerve roots (Chung model neuropathy), partial nerve ligations (Shir model) or nerve compressions (Bennett model) [133,134], and poly-neuropathies may arise from toxic effects from chemotherapy (vinca alkaloid, platin drugs) [135] or from diabetes [136]. Mechanistically, these changes in neuraxial function result from the development of ectopic activity in the sprouting terminals of the injured axons and in their DRG [137]. While not necessary for afferent conduction, DRG activation can be a robust source of afferent traffic into the dorsal horn [138]. This increased activity results from a number of neuro-inflammatory responses which contribute to development and maintenance of the neurogenic pain state: i) increased DRG transcription factor activity [139] associated with altered channel (e.g., increased sodium/decreased potassium) receptor (e.g., increased purine, glutamate) protein expression [140,141,142]; ii) migration of inflammatory cells into the DRG that release lipid mediators, cytokines and growth factors which can activate the neighboring sensory neurons [143,144,145]; iii) sprouting of sympathetic terminals into the neuroma and DRG, which can activate afferent input [146]; iv) activation of DRG satellite cells (glia) [144,147], and development of an enhanced excitatory coupling between these satellite cells and dorsal root ganglion neurons [148], reflecting increased trafficking of GAP junction and excitatory purine receptor expression [149]. Following nerve injury, the increased ongoing afferent input can lead to a central facilitatory response, as observed after tissue injury [150]. In addition, nerve injury may lead to changes in spinal processes resulting in a loss of inhibitory control over large afferent-evoked excitation that likely lead to the phenomena of enhanced sensitivity to light touch (tactile allodynia) [151,152]. Unlike the states associated with tissue injury, pain secondary to injury of the peripheral nerve rarely resolves, resulting in chronic pain states. 

#### 3.3.2. Botulinum Toxin

In rodent models, intraplantar delivery of BoNT/A1 and BoNT/B1 in doses of 0.5–1 U has been reported to reduce the allodynia observed in mononeuropathy models, including those of peripehral nerve ligation [84,85], ventral root transection [86,87], and infraorbital nerve constriction [74], and in polyneuropathy models, including those produced by chemotherpeutics [78,84] and diabetes [88]. As with the models of inflammation, these effects begin after ~1–2 days and last in excess of 21 days. In human case reports and clinical studies, the hyperesthesia observed in post-herpetic neuralgia [49,50,51], diabetic neuropathy [52], and peripheral nerve injury [41,42,43] is relieved by peripheral BoNT/A1 treatment. The use of local intramuscular, subcutaneous delivery of BoNT/A1 has also been reported to have efficacy in trigeminal neuralgia [44,45,46,47,48], which is a facial mononeuropathy. These data suggest that BoNT/A1 and /B1 can reduce neuropathic pain states resulting from peripheral nerve injury. 

## 4. Effects of Peripherally Delivered Botulinum Toxins on Nociceptive Linkages

As reviewed in Section 3 above, peripherally delivered BoNT/A1 and /B1 have been studied in mouse and rat behavioral models and in human pain states for their effects upon several mechanistically defined nociceptive states. Initial work quite reasonably hypothesized that the efficacy of peripherally delivered BoNT/A1 in treating some pain conditions, such as hypertonic states (e.g., torticollus and back spasm), was secondary to muscle relaxation. This hypothesis, however, did not generate continued support, as pain alleviation in conditions like cervical dystonia was noticed much earlier than the muscle relaxation [9], and analgesic efficacy was observed in pain states where muscle tension was not considered to be a primary issue, such as in migraine [9,153] or neuropathic pain states [154]. Current evidence suggests several possible local and spinal mechanisms of action, which might account for the behaviorally defined antinociceptive actions of the peripherally delivered BoNT/A1 and /B1. The effect of BoNT/A1 and /B1 on these pain states reflects upon the systems with which they interact and the cellular effects in these systems. The primary effect of the BoNT toxins is to cleave SNAREs. SNAREs are densely localized in the lipid rafts that organize transport of specific proteins such as receptor/channel subunits and enzymes to the cell membrane of a variety of cell types including neurons and glia [155,156,157,158,159,160]. Thus, while the role of SNARES in mobilizing synaptic vesicles is well known, there are other intracellular transport processes that employ these SNARE targets, and their cleavage would thus impact upon other cellular processes [90,161,162,163,164]. Many of these specific processes are known to mediate nociceptive processing at the level of the primary afferent and the spinal dorsal horn [90,165] and studies supporting these mechanisms are summarized in Table 3. These components and their contributions to the analgesic effects of peripherally delivered BoNTs will be considered below. 

### 4.1. Local Action on Peripheral Afferent Terminal

**Figure 3:**
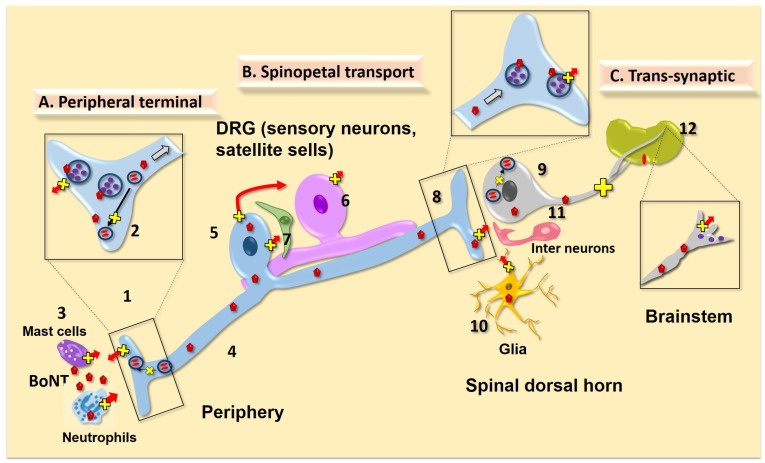
Schematics of possible mechanisms of action of peripherally applied BoNT/A1 and /B1 upon nociceptive processing: (**A**) *Periphery:* (1) At the site of injection BoNTs are endocytosed into the local peripheral afferents, where they cleave SNAREs, thereby inhibiting vesicular fusion and exocytosis of neurotransmitters. This in turn would block vasodilation, plasma extravasation, and activation local inflammatory cells. (2) BoNTs may also regulate SNARE-mediated cell surface expression of a variety of receptors and channels implicated in peripheral sensitization (e.g., TRPV1). (3) Another hypothesis is that BoNTs may enter local resident cells (e.g., mast cells) or migrating cells (e.g. neutrophils) otherwise evoked by injury or inflammation and may directly block the release of cytokines or pro-inflammatory molecules. These release products can activate and sensitize local small afferent terminals. (**B**) *Spinopetal transport*: (4) Following endocytosis in the peripheral terminals, some of the BoNT appears to undergo retrograde transport along the axon. (5) These transported BoNTs reach the dorsal root ganglion (DRG) neuron and cleave SNAREs in the DRG neurons to block vesicular release of neurotransmitters into the extracellular milieu of the DRG, which would otherwise activate and (6) excite the neighboring sensory neurons or (7) closely associated satellite cells. (8) BoNT may further undergo intra-vesicular axonal trafficking to the central terminals, where again by truncating respective SNAREs, it would inhibit neurotransmitter release, thereby preventing the activation of second order neurons and neighboring glial cells. (**C**) *Trans-synaptic actions*: BoNTs can undergo long axonal transport in intact form in non-acidic endosomes and may possibly undergo transcytosis centrally either to the (9) second order neurons or (10) glial cells. Activated glial cells release a plethora of pro-algesic substances (cytokines, chemokines, lipids, amino acids) serving to initiate and maintain central sensitization. (11) BoNTs may also get transcytosed to excitatory (glutamatergic) or inhibitory (GABA/glycinergic) interneurons and may act to block their neurotransmitter release resulting in a loss of excitatory drive or inhibitory control, respectively). Cleaved SNAREs in the second order neurons may interfere with fusion of endosomes that carry the receptors to the membrane. (12) Though speculative, if there is transcytosis to second order projection neurons, it is a reasonable hypothesis that these BoNT are transported to distal terminals which lie in the brainstem and further block the neurotransmission into the brainstem and higher centers.

There is strong and diverse evidence that, as shown in Figure 3, locally delivered BoNTs can be taken up in the terminal of the sensory afferent, and local effects exerted by this toxin will be considered below. 

#### 4.1.1. Afferent Terminal Uptake/Transport

BoNTs can be taken up in the peripheral terminal of motor axons to cleave terminal SNAREs and block release of acetylcholine. It is now evident that BoNT/A1 and /B1 can also be locally taken up at peripheral terminals of sensory axons. In addition to *in vitro* work showing that BoNTs (primarily BoNT/A1/B1) can enter and cleave SNAREs in all neurons and not just motor neurons (reviewed in [166]), principal support for this hypothesis of a terminal action in sensory neurons is derived from the following observations: 

i) Activation of the peripheral terminals of TRPV1 (+) C-fibers with capsaicin leads to the local vesicular release of neuropeptides such as SP and CGRP. This release results in vasodilation and plasma extravasation (e.g. neurogenic inflammation) [167]. In rodents, unilateral subcutaneous BoNT/A1 and /B1 injections into the paw at intervals as short as 15 min to 1 h will block the ipsilateral, but not contralateral, flare and plasma extravasation otherwise evoked by bilateral intraplantar injections of capsaicin. This indicates a local block of the release of these small afferent peptides after local BoNT/B1 treatment [67,75] (Figure 3, #1). 

ii) A block of dural extravasation (from trigeminal afferents innervating the meninges) has been similarly demonstrated [74]. 

iii) Consistent with these findings, human studies also showed similar effects of BoNT/A1 in preventing local cutaneous flare and/or extravasation [57,58,91], though negative results have been reported [61,62,63]. The positive results are consistent with the ability of toxins to prevent afferent transmitter release at the peripheral terminal.

#### 4.1.2. Effects of Local BoNTs on Peripheral Non Afferent Cell Systems 

Locally delivered BoNTs may also affect mechanisms in inflammation other than the afferent terminal (Figure 3, #3). Neutrophils, macrophages and mast cells, when activated, will release local inflammatory products that activate small afferent terminals [168,169], and all employ SNAREs in their transport and exocytosis [170,171]. Macrophages employ a constitutive secretory pathway for the secretion of cytokines (e.g., TNF; IL-6 and IL-10), which mediates trafficking to the cell surface by SNARE-dependent membrane fusion [172]. Neutrophils store pro-inflammatory cytokines preformed in granular packaging and release a variety of highly cytotoxic and stimulatory products including myeloperoxidase and a variety of matrix metalloproteinases through a variety of SNARE- mediated processes [171,173,174]. Mast cells play a major role in the local inflammatory response and their degranulation leads to the local release of a variety of products, including histamine, high molecular weight heparins and cytokines through differential release of granule proteins and distinct secretory pathways, and other dilatory products that are similarly mediated by a variety of SNAREs [175] (Figure 3, #2). These SNARE dependent secretory processes [176,177] are potentially sensitive to effects by BoNTs, provided that the BoNT can enter the relevant cells and cleave a SNARE involved in such secretory processes. Entry of BoNTs into mast cells has not been demonstrated so far and the main SNAP isoform expressed in human mast cells is the BoNT/A1 insensitive SNAP-23. In addition, it appears that VAMP8, rather than the neuronal and BoNT sensitive VAMP1/2, is involved in the secretory process of mast cells [177]. However, local BoNT/A1 has been reported to reduce mast cell degranulation in rodents [178] and reduce burn-induced itching in humans [179]. Although involvement of SNARE proteins in the release of mediators from neutrophils and macrophages has been demonstrated, the direct effect of BoNT/A1 on this release thus far has not been adequately explored. Further research efforts are warranted to determine whether and how BoNTs may have an effect on neutrophils, macrophages and mast cells, and the role that they play in altering the local injury milieu. 

#### 4.1.3. BoNT Effects Upon Sensitized Afferent Terminals 

In studies with dural C fiber afferents, chemical sensitization results in an enhanced afferent response [180]. This facilitated response is prevented by local BoNT/A1 [181]. Further, BoNT/A1 prevented the development of mechanical hypersensitivity of these afferents. Whether this effect reflects a direct effect upon mechanisms underlying the initiation of an excitable state in the afferent terminal (e.g., mobilization of protein kinases leading to phosphorylation of terminal channels/receptors) [168,169], or a potential block of release of active factors from local inflammatory cells (e.g., mast cells, macrophages and lymphocytes) remains to be determined (Figure 3, #3). In short, these findings emphasize that, overall, while BoNT/A1 may not directly alter the acute activation of afferent terminals (e.g., absent effect upon normal sensory thresholds), the toxin can be taken up in an active form in the homologous (ipsilateral) sensory afferent terminal and potentially in local pro-inflammatory cells to reduce the release of local pro-excitatory products otherwise mobilized by injury and inflammation. 

#### 4.1.4. Trafficking of Receptor Subunits in the Primary Afferent and DRG/TG

SNAREs are localized in the lipid rafts [182] which play a role in the organization of membrane protein insertion [183]. Cleavage of SNARES in DRG/TG neuronal cell culture has been reported [92,93,94,95,184] and therefore, it would not be surprising if BoNTs had robust effects upon the trafficking of a variety of membrane proteins. One example would be the TRPV1 channels [185]. It has been shown that during hyperpathic states, TRPV1 receptor function is enhanced by increased trafficking of that protein to the plasma membrane at the peripheral and, likely, central terminals and the DRG. Evidence suggests that BoNT/A1 hinders recruitment of TRPV1 by affecting regulated exocytosis evoked by capsaicin in the trigeminal system [90]. This paradigm suggests a mechanism by which BoNT/A1 would be effective in attenuating hyperalgesia in capsaicin-induced trigeminal sensitization in humans [58] (Figure 3, #2). Other membrane proteins, the trafficking of which is relevant to facilitated pain states, warrant further study, including those for the glutamate ionophores [186] and the TRP channel [187,188]. 

#### 4.1.5. Role of Local (peripheral) BoNT Action in Spinal Nociceptive Transmission

While it is clear that BoNT/A1 and /B1 can exert an effect upon local terminal function, as reviewed above, the majority of work to date has not supported a direct effect of these BoNTs on peripheral terminal excitability in the normal state, as measured by baseline thermal and mechanical thresholds. As noted above, however, it was reported that BoNT/A1 inhibited responses to mechanical stimulation of the dura but only to supra-threshold forces [181]. These electrophysiological studies suggested that BoNT/A1 is selective for C- but not Aδ-trigeminal meningeal nociceptors. That report is consistent with the observation that BoNTs can block release secondary to C-fiber activation, e.g., intraplantar BoNT/A1 or /B1 block capsaicin-induced flare/plasma extravasation in animal [67,74] and human models [58,59,60,132], reflecting the local stimulatory effects mediated by the TRPV1 receptor on the peripheral terminals of the somatic peptidergic C-fiber. While these BoNTs have relatively little effect upon normal pain thresholds, they are noteworthy for their action on facilitated states as they occur after local inflammation and nerve injury. As reviewed above, local BoNT/A1 and /B1 can block the facilitated pain response generated by a variety of stimuli such as intraplantar irritants (e.g., capsaicin, formalin, histamine), but it is not apparent whether this peripheral action accounts for the “analgesic” actions of local BoNT/A1. Several points should be noted. While BoNT/B1 can have an acute onset at the terminal as measured by plasma extravasation after local injection, the effect, for example, on formalin-evoked flinching parallels the reduction in DRG SNAREs, which appears to require a longer interval (e.g., 24 h) [67]. Further, in the case of the formalin flinching model, local BoNT/A1 or /B1 have little or no effect upon the first phase flinching but produce a long-term reduction in the second phase, which is believed to reflect upon a centrally-mediated facilitation of spinal function generated by the conditioning discharge observed during the first phase [67,68,69,70,71,72,73,189]. These results suggest that local BoNT/A1 and /B1 may alter, with short latencies, the processes leading to changes in transduction and the discriminable process of terminal release, thereby preventing pain transmission to the second order neuron. There may be additional mechanisms that are at play and are reflected in part by the delayed onset of effects that are not local but mediated by transport from the site of delivery. 

### 4.2. Spinopetal Afferent Transport 

Most pharmaceutical applications of BoNT/A1 and /B1 are based on the premise that BoNTs remain and act locally at the injection site. However, recent years have brought to light evidence indicating that BoNT/A1 and /B1 may in fact be retrogradely and anterogradely transported after local uptake into terminals. While still controversial, such potential transport of BoNTs is important to consider when determining their mechanism of action in blocking nociceptive transmission (Figure 3, #4). The different lines of evidence supporting spinopetal afferent transport are discussed below. 

#### 4.2.1. Evidence of Transport of Active BoNT in the Primary Afferent

Several lines of evidence now indicate that, apart from a local action, BoNT serotypes A1 and B1 are taken up and transported in the neuron to distal terminals. Fast axonal transport for BoNT/A1 has been demonstrated in the brain [190,191,192]. However, in contrast, a slow movement of BoNT/A1 was reported as cleaved SNAP-25 appeared along neurites and accumulated in the soma over several weeks [193]. In the primary afferent, such transport after subcutaneous/intramuscular BoNT/A1/B1 delivery has been suggested by data derived from several experimental strategies.

i) Movement of radiolabeled (iodinated) BoNT/A1 and /B1 has been reported in spinal pathways [194,195,196]; 

ii) Cleavage of SNARE proteins (SNAP-25-BoNT/A1 / VAMP-BoNT/B1) has been detected in the ipsilateral afferent cell body (DRG/or trigeminal ganglia (TG)) after intraplantar delivery [67,71,197,198]. BoNT/A-cleaved SNAP-25 appeared bilaterally in the ventral and dorsal horns four days after injection of BoNT/A1 / BoNT/A2, suggesting that catalytically active BoNT/A1 and BoNT/A2 were transported via peripheral motor and sensory nerves and transcytosis [199]. 

iii) A block of dorsal horn substance P release from primary afferents in the nucleus caudalis evoked by supraorbital capsaicin [75] or in the spinal dorsal horn evoked by intraplantar formalin has been observed after BoNT/B1 treatment [67]. Further, the same authors showed that peripherally delivered BoNT/B1 blocked the evoked release of substance P in the ipsilateral but not contralateral dorsal horn (otherwise evoked bilaterally by spinally delivered capsaicin). This demonstrated that the BoNT/B1 delivered peripherally to one paw had to reach the ipsilateral central afferent terminals where the intrathecal capsaicin was acting.

iv) BoNT/B1 treatment resulted in a block of evoked dorsal horn activity as measured by a decreased incidence of evoked dorsal horn c-Fos (+) neurons [67,75]. These observations indicate that peripherally delivered BoNT/B1 is taken up at the peripheral afferent terminals and transported in an active form to central afferent terminals in both spinal and trigeminal systems, where they cleave SNAREs and prevent transmitter release (Figure 3, #5, #8).

v) Retrograde and anterograde transport of BoNT/A1 in non-acidic organelles has been shown by several groups, and estimates indicate speed profiles matching fast microtubule-dependent transport that overlaps with TeNT (tetanus neurotoxin) positive carriers in motor neurons and *in vivo* models [74,89,190,191,192,197,199,200,201,202]. However, it is currently unclear what fraction of the locally administered BoNT/A1 undergoes such intraneuronal transport, and whether the transport is concentration-dependent or differs mechanistically for different routes of administration.

The likelihood that BoNT/A1 and /B1 transported centrally in the afferent can block transmitter release is an important component of understanding their antinociceptive action. However, a common thread in the actions of the BoNTs on pain behavior is the general lack of effect upon acute nociceptive thresholds. Given the role of small afferents in pain transmission, it is puzzling that blocking central terminal release fails to alter acute transmission (if not producing an all-out anesthesia). This conundrum is not limited to the action of BoNTs. Studies with intrathecally delivered calcium channel blockers (e.g., for the N-type channel) showing effects on afferent transmitter release typically fail to block large afferent input (light touch) and have little effect upon acute pain thresholds, yet BoNT/A1 and/B1 delivery prominently reduces facilitated states in animal models and in humans [203,204,205,206,207,208,209]. The reason for this apparent discrepancy remains to be characterized. In short, however, the evidence for the time-dependent central transport of BoNT/A1 in an active form in the primary afferent and cleavage of SNAREs in the cell body (DRG/trigeminal ganglion) emphasizes the potential for a centrally mediated change in transmitter release from the central afferent terminal and, perhaps less appreciated, from its cell body (DRG/trigeminal ganglion) [126]. 

#### 4.2.2. Role of Cell Body (DRG) Release and BoNT Action

As noted above, transmitter release of glutamate, among other transmitters, occurs in isolated DRG/trigeminal ganglion cells and from associated glia (satellite cells) in cultured cells [96,210,211]. Such release likely also occurs *in vivo* [212]. Electrophysiological recording studies have indicated cross-excitation between large (low threshold tactile sensitive) afferents and small (high threshold, nociceptive) afferents. This interaction, potentially leading to tactile evoked activation of nociceptive input, is enhanced after peripheral injury [213,214,215]. Current work strongly suggests that such release could indeed activate other DRG cells through, for example, glutamatergic receptors [216], some of which are on DRG glia (satellite cells) [217] (Figure 3, #6, #7). Given the contribution of such DRG release to hyperpathic states initiated by nerve and tissue injury, along with the ability of peripherally injected BoNT/A1 and /B1 to cleave DRG and trigeminal SNAREs, it is an intriguing hypothesis that such an action by BoNTs could be responsible for their modulation of primary hyperpathia and large afferent mediated allodynia. Recent work has indeed shown that BoNT/A1 decreases vesicular release of glutamate from satellite cells [96]. A recent study importantly suggests that BoNT/A1 may interfere with intra-ganglionic transmitter release and cross-excitation of neighboring neurons to the development and maintenance of chronic pain states [218]. This transported action might thus provide an important mechanism for altering the hypothesized crosstalk between populations of sensory ganglion cells (Figure 3, #6, #7). Further studies in this area will be of considerable interest. 

#### 4.2.3. Effect of BoNT on Central Trafficking

As reviewed above, SNAREs play a ubiquitous role in the trafficking of several ionotropic and metabotropic receptors that contribute to regulating neuronal excitability and synaptic transmission in the dorsal horn. The protein receptors are transported to the cell surface in the vesicle membrane that fuses via a SNARE mediated process. SNAREs thus play a major role in the induction and maintenance of spinal sensitization. SNARE proteins are involved in the mobilization of receptor subunits of AMPA [219] and NMDA [220], which are actively involved in the process of dorsal horn sensitization [221,222,223]. Interventions by BoNT in glutamate ionophore trafficking would influence facilitated processing, thereby having profound effects upon the generation of facilitated states. In cerebellar slices, BoNTs reduce trafficking and block long-term potentiation [161]. The potential that BoNTs may affect the processing of pain information by altering receptor trafficking in addition to direct effects resulting from a block in exocytosis is an interesting future area of study.

#### 4.2.4. Effect of Intrathecally-Delivered BoNTs 

Consistent with these spinal actions outlined above, not surprisingly, intrathecal delivery of BoNT/B1 has been shown to result in cleavage of spinal SNAREs, blockade of evoked release of afferent transmitters and prevention of evoked activation of spinal neurons as indicated by the incidence of c-Fos (+) neurons [67,224]. Intrathecal delivery of BoNT/A1 and /B1 has been shown to block the second phase of IPLT formalin-evoked pain behavior [67,73,225] to reduce visceral-evoked inflammatory states (BoNT/A1) [226], and to attenuate the allodynia otherwise observed in mononeuropathies (nerve ligation) and polyneuropathies (as with chemotherapeutics) (BoNT/B1) [84]. The specificity of these effects on the role played by the cleavage of SNAREs was provided by two observations: i) intrathecal delivery of BoNT/B1 treated with dithiotreitol to cleave the disulfide linkages had no activity, and ii) intrathecal BoNT/B1 serotype blocked formalin-evoked flinching in the mouse but not in the rat, an observation consistent with the fact that the rat VAMP1 has a mutation at the BoNT/B1 targeted cleavage site rendering the rat insensitive to BoNT/B1 activity [227]. 

While several lines of evidence indicate that intrathecal BoNT/A1 and /B1 can affect afferent terminal function, as reviewed above, they may also be taken up by a variety of neural types including inhibitory interneurons (Figure 3, #11). Cell culture studies have shown that several BoNTs can block depolarization-evoked release of inhibitory amino acids [92,228,229,230]. A block of spinal inhibitory interneurons would lead to excitation, and such has been reported after accidental intrathecal delivery of BoNT/A1 in humans [231]. This is a very important point of consideration when evaluating BoNTs as pain pharmaceuticals.

### 4.3. Trans-Synaptic Effects

While the axon transport of active BoNT/A1 and /B1 appears evident, it has long been argued that the BoNTs, unlike tetanus toxins, do not undergo trans-synaptic movement. However, current diverse findings from several research groups lend support to such a possibility, such that movement to second order neurons and glia appears to be a tenable hypothesis (Figure 3, #9#10). Several examples will be noted.

i) Direct evidence supporting the hypothesis that BoNT/A1 is retrogradely transported in an active form and undergoes transcytosis into second order neurons was provided by the detection of BoNT/A1 cleaved SNAP-25 in tectal synapses after injection of the neurotoxin in the rat eye [191]. 

ii) In the trigeminal system, BoNT/A1 injected into the temporomandibular joint inhibited plasma extravasation in the dura mater, indicating the likelihood of trans-synaptic movement from mandibular afferents to meningeal afferents [74]. 

iii) Similarly, in the trigeminal system, BoNT/B1 injected in the supraorbital region blocked substance P release (as measured by NK-1 receptor internalization) and c-Fos activation in the ipsilateral nucleus caudalis evoked by meningeal capsaicin. This indicates an effect of the subcutaneously delivered BoNT/B1 on meningeal afferents [75]. 

iv) Cleavage of SNAP-25 in the glial cells of the spinal cord dorsal horn has been observed following injection of BoNT/A1 in the paw [232]. 

v) Activation of c-Fos can be initiated bilaterally by intrathecal sP through an action on NK1 receptors. Following unilateral intraplantar BoNT/B1, the ipsilateral (but not contralateral) dorsal horn c-Fos activation is reduced [67]. As the receptors for sP (NK-1R) are postsynaptic to the primary afferent [233], ipsilateral block by IPLT BoNT/B1 after bilateral activation is considered to represent an effect postsynaptic to the primary afferent terminal. 

vi) Subcutaneous BoNT/A1 reduced the number of c-fos activated neurons following intracisternal NMDA injection (Kim et al., 2015) and therefore suggests a trans-synaptic effect. Whether functional NMDA receptors are present only post-synaptic is a topic of discussion.

vii) In rats, a BoNT/A1 and BoNT/A2 serotype injected into a forelimb was observed to produce a contralateral weakness, suggesting that the BoNT traveled retrogradely and trans-synaptically to contralateral spinal motor neurons [125]. 

viii) In humans, BoNT/A1 was found to reduce recurrent inhibition of motor neurons after intramuscular BoNT/A1, indicating an effect upon an inhibitory interneuron, consistent with a trans-synaptic movement from the motor neuron [202]. These results jointly provide a broad spectrum of data in multiple neuraxial systems suggesting that BoNT/A1, B1 and A2 are not only transported to the central terminal, but may also undergo trans-synaptic movement from the central afferent terminal to second order neurons and glia. 

### 4.4. BoNT Actions on Glial Cells

As noted above, DRG satellite cells can release glutamate in a SNARE-dependent fashion and likely play a role in regulating local excitability. Similarly, activation of dorsal horn glia and interactions between neurons and glia are considered to play a major role in facilitated pain states [234]. Astrocytes (and microglia) express a full complement of SNAREs and these regulate their exocytotic activity for peptide and amino acids (glutamate) [74,232,235,236,237]. While some report that astrocytes do not express SNAP-25 and instead express BoNT/A1 resistant SNAP-23 [238,239,240], others demonstrate a decrease in neurotransmitter release from astrocytes after BoNT/A1 treatment [241]*. In vivo* work suggests that astrocytes indeed express SNAP-25 [232,242]. Given the role of glial activation in dorsal horn sensitization**,** regulation of their functionality would powerfully regulate dorsal horn facilitated states, as after nerve and tissue injury. While currently speculative, this is an important potential aspect of pain regulation by BoNTs that requires further exploration (Figure 3, #10).

## 5. Diversity of Botulinum Neurotoxins

Almost all of the above studies have been conducted on the two BoNT subtypes that are currently marketed as pharmaceuticals, BoNT/A1 and B1. These studies help in elucidating the mechanisms that are involved in inhibition of pain states by BoNT/A1 and /B1. Armed with this constantly increasing knowledge base, it is intriguing to consider discovery or design of novel BoNT based pharmaceuticals with characteristics that will lead to improved targeting of nociceptive pathways. As reviewed above, BoNTs are classified into seven immunologically distinct serotypes (A–G). In addition to the well- characterized and defining immunogenic differences [29], the serotypes can also be functionally distinguished by several properties.

**Table 3:** Key findings supporting antinociceptive actions of BoNTs after local injection.

No	Mechanism of Action	BoNT Serotype	Species/Models	Interpretations	References
1)	Local Peripheral effect	BoNT/A1/B1	Human/rat/mouse	Blocks local flare, vasodilation and plasma extravasation evoked by local irritants in human and animal studies	[57,58,59,60,66,67,74,91,132]
			Human/rat/mouse	Lack of effect on normal sensory thresholds. For e.g. acute thermal and mechanical pain thresholds	[57,61,62,63]
			Rat/mouse	Effective only in facilitated pain states such as activation of c-fibers. Does not affect phase I of formalin flinching with significant effects on facilitated phase II	[67,68,69,70,71,72,73,117,181]
2)	Axonal transport	BoNT/A1/B1	Rat/Cell culture	Fast and slow long distance axonal transport in neurons	[190,191,192,193]
			Cat/rat	Movement of radiolabeled toxin observed in motor and sensory pathways. However, transport of radiolabeled isotope *per se* were not evaluated	[194,195,196]
			Rat/mouse	Cleavage of SNARES in cell body/soma (DRG/TG) *i.e*., away from the site of BoNT injection	[67,75,197,198]
			Rat/mouse	Cleavage of SNARES in the central terminals in spinal cord following peripheral BoNT	[67,197]
3)	Central actions	BoNT/A1/B1	Rat/mouse	Inhibits substance P release evoked by intrathecal capsaicin in spinal cord following peripheral BoNT. Blocking neurotransmitter release after retrograde transport. Inhibition of nociceptive behavior and neuronal activation of c-fos	[67,192]
		BoNT/A1	Rat	Blocking the axonal transport of peripheral BoNT in trigeminal and sciatic nerve using colchicine inhibits hyperalgesia	[71,74,89,197]
			Rat	Bilateral effect of unilateral BoNT in diabetic neuropathy and trigeminal neuropathy	[74,78,88,89]
4)	Trans-synaptic actions	BoNT/A1	Rat	BoNT is transported from retina to colliculus and transcytosed to tectal synapses as observed by SNARES cleavage	[191]
		BoNT/A1/B1	Rat/mouse	Peripheral BoNT inhibited intrathecal substance P induced and intracisternal NMDA induced neuronal activation.	[67,243]
			Rat/mouse	Supraorbital BoNT reduced meningeally evoked activation of second order neurons and substance P release. BoNT in TMJ inhibits dural plasma extravasation	[74,75]
		BoNT/A2	Rat	Peripheral BoNT cleaved SNARES in spinal glial cells	[232]
		BoNT/A1	Rat	Unilateral BoNT produce contralateral muscle weakness and bilateral SNARE cleavage	[125,190]

### 5.1. SNARE Target and Cleavage Sites

Each BoNT serotype has its own unique SNARE cleavage site. BoNT/A and E both cleave SNAP-25 at distinct peptide bonds. BoNT/C also cleaves SNAP-25 at a distinct site and additionally cleaves syntaxin [34]. BoNT/B, D, E, and F all cleave synaptobrevin 1 and 2 (VAMP), each at different peptide bonds. While the subtypes within one serotype generally all cleave the same SNARE at the same peptide bond, two exceptions are known. BoNT/F5 and a BoNT/FA hybrid toxin [244,245,246] have a unique VAMP1/2 cleavage site [246,247]. 

### 5.2. Membrane Sites Mediating Internalization

Another very important consideration is that there are membrane sites that mediate the internalization of the BoNTs. While for several BoNTs the membrane receptors have been identified, they have not been established for all serotypes, and certainly not for all subtypes. All BoNTs seem to associate with gangliosides, and specific protein receptors have been identified for several BoNTs (synaptotagmin I and II for BoNT/B1 and G, and SV2 for BoNT/A1, D, and E1) [248]. Until recently, the SV2 receptor was considered to be the only protein receptor required for BoNT/A1 cell entry in conjunction with ganglioside binding [249]. Recently, Fibroblast Growth Factor Receptor 3 (FGFR3) has been suggested to also be critical for BoNT/A1 uptake. Overexpression of FGFR3 correlated with increased BoNT/A1 entry [250], although it is not clear whether the effect is direct or indirect. A direct involvement of FGFR3 in BoNT/A1 cell entry might have important implications in the use of this toxin as a pain therapeutic. DRGs (e.g., primary afferents) constitutively express FGFR3 and show up-regulation after injury [251,252]. FGFR3 is also expressed in astrocytes and microglia, and this expression is upregulated by afferent drive [253,254]. Thus, if increased expression of FGFR3 aids BoNT/A1 cell entry, persistent afferent activation may lead to an increased up-take of BoNT/A1 into DRGs, astrocytes and microglia. This topic requires further exploration. 

### 5.3. Species Specificity 

Given these distinctions in cleavage sites and membrane binding sites, it is not surprising that there are differences in species specificity. BoNT/A, B, E, and F cause human botulism, whereas C and D cause botulism in animals, and G is usually not associated with botulism [1]. Rats, but not humans or mice, have a single amino acid polymorphism in its VAMP1 sequence that renders it resistant to BoNT/B1 [227]. Humans and some rodent species have a polymorphism in VAMP1 that renders it a less efficient substrate for BoNT/D [255,256,257]. In addition, the synaptotagmin II receptor in humans and chimpanzees was found to be a weak receptor for BoNT/B1, D-C and G [258,259], which incidentally explains the higher dose required for rimabotulinumtoxinB (pharmaceutical BoNT/B1) compared to pharmaceutical BoNT/A1. 

### 5.4. Duration of Action

Serotypes and even subtypes within one serotype may vary in their duration of action [35,260], reflecting differences in intracellular half-life of the LC [261,262,263,264]. In humans, durations rank in parallel with those noted *in vitro* and in mice [263,264]: e.g., local intramuscularly delivered BoNT serotypes A, B, C, E and F cause local paralysis lasting about 3–6, 3, 2–4, 1–3, and 1–2 months, respectively [261,262,264,265,266,267,268,269]. BoNT/D was poorly effective in causing local muscle paralysis in humans [270], and duration of action in mice was significantly shorter than that of BoNT/A1 [271].

Given the above distinctions, it is clear that significant differences exist within the serotypes and the subtypes for a given serotype that may affect the pharmacological properties of these toxins. Only a few studies so far have analyzed subtype-specific characteristics, and future efforts in that regard will no doubt reveal BoNTs with unique properties. Recent studies comparing BoNT/A1–5 have revealed significant functional differences between these toxin subtypes, including differential sensitivity of various cell-based models, differences in enzymatic activity, differences in symptoms in mice after injection of high toxin doses and differences in onset and duration of action [35,272,273,274,275]. For example, injection of mice with high doses of BoNT/A2 and A3 resulted in an overall increasing paralysis, whereas injection with BoNT/A1 and A5 caused typical botulism symptoms, such as ruffled fur, wasped waist, difficulty breathing and spasticity just prior to death [272,275]. In addition, the few studies that have been undertaken to analyze the cell surface receptors for BoNT subtypes within one serotype indicated significant differences [274,276]. These data, in combination with differential sensitivity of cell models to these toxins [272], may indicate unique pharmacologic properties of subtypes within one serotype**.** In addition, BoNT/A3 was shown to have a shorter duration of action than BoNT/A1, 2, 4 and 5 [35,277], whereas BoNT/A2 was shown to enter neurons faster than BoNT/A1 and to be more potent *in vivo* in causing muscle paralysis and central effects [69,125,199,272,274,278,279,280,281,282,283]. Catalytic activity of a few subtypes has also been compared, and these studies have revealed that subtypes within one serotype can have distinct substrate binding and cleavage properties [272,273,284], and in the case of BoNT/F5 can even have a unique substrate cleavage site [247]. 

An initial attempt at redesigning a BoNT with unique pharmacological properties has resulted in the creation of BoNT/AE (A1 LC and HC translocation domain, E1 HC receptor binding domain) and BoNT/EA (E1 LC and HC translocation domain, A1 HC receptor binding domain) chimeric constructs [285]. All constructs resulted in potent muscle paralysis in mice, and the onset and duration of action and cell entry properties of these chimeric toxins corresponded to the respective LC-HC-translocation domain fragments. Thus, the EA chimera displayed a faster onset of action and shorter duration similar to BoNT/E1, whereas the AE chimera had a slower onset and long duration of action similar to BoNT/A1. Interestingly, while BoNT/E appears to be unable to bind to and enter trigeminal ganglion neurons (TGNs), the EA chimera was found to enter trigeminal ganglion neurons (TGNs) and cleave SNAP-25, leading to inhibition of CGRP release, a pain signaling peptide [229]. This study, while not directly providing a novel pain-specific therapeutic, demonstrates proof-of-principle that different domains of BoNTs can be combined to create unique BoNT based constructs that allow novel neuronal targeting. 

Further research is required to fully elucidate the diversity of this large family of protein toxins, and to determine the structural motifs responsible for a particular functional property. Structural studies of BoNTs are steadily advancing, and since the first elucidation of the crystal structure of BoNT/A1 in 1998 [36], high resolution structures of several other serotypes and functional domains have been reported 6 analyses have identified amino acid residues required for this binding [286]. These are significant advances that, with further detailed studies of other BoNTs and receptor complexes, will eventually lead to a better understanding of the structure-function relationships of these toxins and enable design of safer and targeted pharmaceuticals. 

## 6. Future Directions in Botulinum Neurotoxins as Pain Pharmaceuticals

The complexity of the BoNT structural motif and the ubiquitous role of SNAREs in neuronal function emphasize that there are numerous directions for discovery in this field. 

### 6.1. Recombinant Technology

Great advances have been made in analyzing the effects of BoNT/A1 and /B1 on pain conditions. However, as discussed above, significantly more research is required to provide a mechanistic understanding underlying the analgesic effects of these BoNTs. Interestingly, there seems to be little difference in the results observed in pain studies using BoNT/A1 or /B1, even though these two serotypes exhibit different characteristics, including different SNARE targets (SNAP-25 for BoNT/A1 and VAMP2 for BoNT/B1), different cell surface receptors and different potencies in causing local paralysis in humans, and BoNT/A1 has a slightly longer duration of action (see Section 5). For other BoNT sero- and subtypes, it is not known whether or how their specific differences impact their effects on nociceptive pathways. Considering the large diversity of BoNTs, analyzing various additional BoNTs for their utility as therapeutics for pain and other disorders is an exciting area of study. Moreover, recombinant techniques in the botulinum neurotoxins field have advanced in recent years to finally enable the expression of recombinant BoNTs in heterologous [276,287,288,289,290] or native [291,292] expression hosts. Such techniques will now allow the design of recombinant BoNT-based constructs that are particularly suited as pain therapeutics. Currently such experiments are labor-intensive, expensive and restricted in several countries including the United States. However, the potentially huge benefits from such studies, such as the development of a pain therapeutic of long duration (weeks to months) without impairing side effects, justify the required effort and resources. For example, it has been shown that a single amino acid substitution in the LC of BoNT/E resulted in a change in SNARE target cleavage, such that the modified LC could efficiently cleave human SNAP-23, which is naturally resistant to cleavage by BoNT/E [293]. This is of particular significance for inflammatory pain, as SNAP-23 seems to be the major SNAP isoform involved in secretory release in mast cells and in glial cells. Of the BoNTs currently identified, several strategies will be cited as examples to further analyze their potential as pain therapeutics. 

### 6.2. Mining through the Diverse Family of BoNTs

Detailed functional and structural studies of the many not yet characterized BoNT subtypes will likely reveal further BoNTs with characteristics that may render them more suitable as pain therapeutics. This involves determining the subtype’s unique functional characteristics and the structural features responsible for these characteristics, and conducting comparative pain studies to determine which of these features would optimize utility of the particular BoNT for pain treatment. Considering the relatively few studies characterizing BoNT subtypes so far, finding such characteristics may seem a bit like the search for a needle in a haystack. While it is certainly exciting to continue to characterize the many BoNT subtypes, more targeted approaches might be possible in the future. By building on the recent progress in structure-function studies and on analyses of factors that influence distribution and the duration of action of these toxins, modeling studies will likely enable the identification of likely candidates or desired structural domains in the future. Based on our current understanding of mechanisms involved in pain processing and BoNTs’ effects on such processes, a few strategies for such a pursuit are listed below. 

i) A BoNT or BoNT derivative that specifically or preferentially blocks transmission of pain fibers while having a lesser effect on motor neurons and inhibitory interneurons would be expected to be a safer alternative to the currently used BoNT/A1 and /B1 for treatment of pain. Due to increased safety, larger amounts of the toxin could be applied, which would lead to greater effectiveness and longer duration of action. Considering the differences in cell surface receptor binding and species specificity among the various BoNTs, future research may identify one or several BoNTs that will preferentially enter or affect nociceptive neurons. Several examples of neuronal cell specificity of different BoNTs can be found in the literature, including differential sensitivity of various neuronal cell models to BoNT/A1-5 [272], unique cell entry properties of BoNT/D in isolated neuronal cultures [271], and differential sensitivity of cultured trigeminal ganglion neurons to BoNT/A1, E1, and chimeras thereof [229]. In addition, specific toxin characteristics may contribute to distinct pharmacokinetic behavior of a particular BoNT subtype, as is evidenced by the observed differences in the pathology and local paralysis symptoms of mice injected with BoNT/A1-5 [272,277] as well as by symptomatic differences in humans suffering from botulism caused by different serotypes [1]. The pharmacokinetics of BoNTs has only been studied for BoNTs that are currently used clinically, and using only a few administration routes [294]. 

ii) As discussed above, BoNTs have been shown to affect receptor trafficking in target cells. This in turn may have profound effects on pain processing. The distinct SNARE cleavage targets and sites of the different BoNTs may affect the fusion of endosomes that carry receptors in distinct ways, and further research needs to be devoted to this aspect of BoNT function with regard to their effect on pain processes. Other factors that may influence receptor trafficking such as intracellular localization also remain largely unstudied. 

iii) The involvement of glial cells in pain processing and the development of chronic pain states has been well established [74,232,235,236,237]. Studying the various BoNT subtypes for their potential effects on glial cells is of particular interest, as the distinct cell binding and entry mechanisms of the different BoNTs raise the possibility that some BoNT types, but not all, may affect glial cells.

iv) For treatment of chronic pain, a long duration of action is desirable. However, in some instances and to prevent the development of (rather than treat) chronic pain, a shorter duration of action (days to weeks) may be more appropriate. The duration of action is highly variable for the different BoNTs, and the mechanisms underlying the persistence of action of BoNTs in neurons are not well understood. Screening of BoNT subtypes for duration of action and pharmacologic properties may detect a BoNT of long duration with the desired pharmacologic characteristics, or alternatively enable construction of a recombinant BoNT combining the functional domains of different subtypes to achieve the same effect. For example, BoNT/A3 has been shown to have a much shorter duration of action in cells and in mice than BoNT/A1 [35,277]. Thus, a subtype of the BoNT most widely used for clinical application but with a shorter duration of action has now been identified. In addition, this subtype exhibits different cell entry kinetics and specificity in various rodent and human cell models [272]. Future studies are required to determine its effect on nociceptive processing.

v) As with current clinical applications of BoNTs, high potency is desirable. Since these toxins are proteins, the formation of antibodies and resistance to further treatments are important considerations. High potency will reduce the chances of such antibody formation as very small amounts of the toxin can be used. 

vi) Toxin distribution and spread from the injection site likely differs for different BoNTs, as has been shown in the case of BoNT/A1 and B1 and BoNT/A1 and A2 [199,278,279]. Toxin spread may contribute to antibody formation and increased side effects. It is important to explore how the administration route affects toxin spread of the various toxins. 

vii) Finally, ease of production, purification and stability of the toxins are obvious important considerations. 

### 6.3. BoNT/C1 and D1

An example of specific directions may be exemplified by the considerations of one specific pair of toxins. *BoNT/*C1 and D1 do not produce human botulism but are responsible for large and devastating animal botulism outbreaks [295,296,297,298,299,300]. Both BoNT/C1 and D1 have recently been shown to be in fact taken up and be enzymatically active in human neurons [266,271,301]. Many reasons could contribute to that, including distribution of the *Clostridium* strains producing these toxins in the environment, uptake of the toxins through the intestinal wall, etc. Interestingly, recent studies of BoNT/D1 have indicated that this BoNT serotype cleaves human VAMP1 less efficiently than VAMP1 of most animals [255,256]. Local injection in humans resulted in a poor paralytic response [270], most likely due to the fact that human motor neurons primarily express VAMP1 [255]. In central neurons, however, differential expression of VAMP1 and VAMP2 has been observed [302]. Thus, BoNT/D1 is an intriguing toxin to study as a pain therapeutic, as it would be expected to preferentially affect sensory neurons expressing VAMP2 over motor neurons, which express mostly VAMP1. Similarly, BoNT/C1 has been shown to enter neuronal cells by a unique pathway, utilizing dual gangliosides as receptors [303]. This unique cell entry pathway is intriguing and analyses of the distribution and pharmacology of this toxin will determine whether it may be a useful candidate as a pain therapeutic. Even though BoNT/C1 is not usually associated with human botulism, local injection studies in humans have shown that it is effective in causing local paralysis [261]. No studies have been conducted assessing the effects of BoNT/C1 on pain transmission. While BoNT/C1 is known to cleave two SNARE proteins, SNAP-25 and syntaxin [304,305], which has previously been proposed to lead to cytotoxicity [306], recombinant technology will now enable selective knocking out of the SNAP-25 or syntaxin cleavage in BoNT/C1 holotoxin, which has already been achieved in recombinant BoNT/C1 light chain constructs [307]. This will create a novel construct that distributes *in vivo* as BoNT/C1 but should not pose the threat of cytotoxicity. The effect of a toxin only cleaving syntaxin on neuronal transmission and, in particular, pain transmission, has never been explored. 

### 6.4. Alternate Targeting Strategies 

The majority of work reviewed in this overview has considered the effects of intramuscular/subcutaneous delivery. Other approaches also lend themselves to delivery of pain therapeutics. 

i) Intrathecal delivery of BoNTs represents an important strategy for affecting central processing of pain input. As reviewed above, several papers have emphasized that action. However, as noted, such an injection may lead to unwanted events because of its broad uptake. An alternate strategy to enhance targeting is coupling the toxin to a ligand for a G protein coupled receptor, which will undergo internalization when occupied, carrying the ligand toxin complex with it. One example of such a construct is substance P-saporin (sP-saporin), which has been shown to produce a prominent analgesia in animals [308,309,310,311,312,313]. However, saporin results in permanent cell death, which is not a desirable outcome for chronic pain treatment. A similar construct utilizing a BoNT LC, with the premise that the LC is not readily taken up in the absence of the HC, would be expected to result in the same analgesia due to a block in transmission of the treated neurons, but the effect would be long-lasting and reversible. One report of such a construct linking substance P to the BoNT/A1 LC did appear to decrease thermal hyperalgesia in animals [314], but further work is required to confirm these data and show specificity. The use of other targeting ligands raises the intriguing possibilities for specific targeting of neuraxial systems. Creation of such targeted secretion inhibitors has been suggested and is pursued by industry [315]. Such targeting peptides may be small peptide transmitters that specifically bind to receptors on neurons involved in pain processing (such as substance P binding to NK1 receptors), or ligands for receptors that are enriched or uniquely displayed on nociceptors, such as the TRPV1 receptors [316]. One such construct containing the BoNT/A1 catalytic function coupled to lectin has been shown to inhibit release of neurotransmitters from DRGs and attenuate sensory transmission from nociceptive afferents [317]. 

ii) Topical delivery to the skin can result in permeation of the large BoNT structure. Transcutaneous movement may be enhanced by several strategies, including the use of synthetic peptides. Such delivery has been shown to result in the movement of active forms of BoNT into the skin with effects on afferent nerve terminals as evidenced by block of neurogenic inflammation [59]. The potential for such a delivery method is strengthened by the observation that cell-permeable forms of a truncated SNAP 25 can penetrate the dermis and competitively inhibit SNARE complex formation [318,319]. The effects of the unique properties of the different BoNTs on such a delivery strategy are unknown. 

## 7. Conclusions

BoNTs comprise a family of protein toxins that are grouped together by their ability to cause botulism in humans or animals. Since the first discovery of immunologically distinct serotypes of BoNTs almost 100 years ago [320], continuing research has revealed the large and diverse nature of this protein family. With advances in sequencing methods, structural and biochemical biology and cell culture technologies, the past 10–15 years in particular have yielded exciting insights into the variety of BoNTs. Now, over 40 different subtypes of BoNTs have been described, and the list is likely to grow longer [295,321]. Only two BoNTs, BoNT/A1 and BoNT/B1, are currently used clinically, mostly for spasticity related disorders and in aesthetics. However, the vast increase in knowledge of this toxin family has now opened the door for exploration of these toxins to treat other neurological conditions, such as chronic pain. While analyzing the currently marketed BoNTs for that application is of high value, the diverse pool of BoNT subtypes provides an additional treasure trove of BoNTs with potentially unique features that may make them more suitable as pain therapeutics than BoNT/A1 and /B1. The most critical question in this pursuit is, ‘What characteristics would render a particular BoNT or BoNT-based construct more suitable as a pain pharmaceutical?’ Only extensive future research on both the mechanism of current clinically used BoNTs on pain processing and the effects of the various BoNT subtypes found in nature on pain processing will address this question. The rewards of such research efforts are obvious and justify the extensive work required to achieve that goal. Further studies on characterizing the BoNT subtypes may also enable the targeted design of recombinant BoNT-based pain therapeutics using specific targeting peptides to direct entry of the toxins into pain transmitting neurons. Most interestingly, analyses of the effects of BoNTs and targeted derivatives on pain processing will at the same time also advance our understanding of the complex mechanisms underlying the encoding of the pain sensation. 

## References

[1] Johnson E.A., Montecucco C., Andrew G.E. (2008). BOTULISM. Handbook of Clinical Neurology.

[2] Drachman D.B., Simpson L.L. (1971). Botulinum toxin as a tool for research on the nervous system. Neuropoisons. Their Physiological Actions.

[3] Scott A.B., Rosenbaum A., Collins C.C. (1973). Pharmacologic weakening of extraocular muscles. Investig. Ophthalmol..

[4] Scott A.B. (1980). Botulinum toxin injection into extraocular muscles as an alternative to strabismus surgery. J. Pediatr. Ophthalmol. Strabismus.

[5] Schantz E.J., Kautter D.A. (1978). Standardized assay for clostridium botulinum toxins. J. AOAC..

[6] Dressler D. (2012). Clinical applications of botulinum toxin. Curr. Opin. Microbiol..

[7] Truong D.D., Stenner A., Reichel G. (2009). Current clinical applications of botulinum toxin. Curr. Pharm. Des..

[8] Pantano S., Montecucco C. (2014). The blockade of the neurotransmitter release apparatus by botulinum neurotoxins. Cell Mol. Life Sci..

[9] Brin M.F., Fahn S., Moskowitz C., Friedman A., Shale H.M., Greene P.E., Blitzer A., List T., Lange D., Lovelace R.E. (1987). Localized injections of botulinum toxin for the treatment of focal dystonia and hemifacial spasm. Mov. Disord..

[10] Whitcup S.M., Turkel C.C., DeGryse R.E., Brin M.F. (2014). Development of onabotulinumtoxina for chronic migraine. Ann. N. Y. Acad. Sci..

[11] Robertson C.E., Garza I. (2012). Critical analysis of the use of onabotulinumtoxina (botulinum toxin type A) in migraine. Neuropsychiatr. Dis. Treat..

[12] Frampton J.E. (2012). OnabotulinumtoxinA (BOTOX^®^): A review of its use in the prophylaxis of headaches in adults with chronic migraine. Drugs.

[13] Dodick D.W., Turkel C.C., DeGryse R.E., Aurora S.K., Silberstein S.D., Lipton R.B., Diener H.C., Brin M.F. (2010). Onabotulinumtoxina for treatment of chronic migraine: Pooled results from the double-blind, randomized, placebo-controlled phases of the preempt clinical program. Headache.

[14] Diener H.C., Dodick D.W., Aurora S.K., Turkel C.C., DeGryse R.E., Lipton R.B., Silberstein S.D., Brin M.F. (2010). Onabotulinumtoxina for treatment of chronic migraine: Results from the double-blind, randomized, placebo-controlled phase of the preempt 2 trial. Cephalalgia Int. J. Headache.

[15] Barrientos N., Chana P. (2003). Botulinum toxin type A in prophylactic treatment of migraine headaches: A preliminary study. J. Headache Pain.

[16] Silberstein S., Mathew N., Saper J., Jenkins S. (2000). Botulinum toxin type A as a migraine preventive treatment. For the BoTOX migraine clinical research group. Headache.

[17] Anand K.S., Prasad A., Singh M.M., Sharma S., Bala K. (2006). Botulinum toxin type A in prophylactic treatment of migraine. Am. J. Therapeutics.

[18] Aurora S.K., Winner P., Freeman M.C., Spierings E.L., Heiring J.O., DeGryse R.E., VanDenburgh A.M., Nolan M.E., Turkel C.C. (2011). Onabotulinumtoxina for treatment of chronic migraine: Pooled analyses of the 56-week preempt clinical program. Headache.

[19] Fadeyi M.O., Adams Q.M. (2002). Use of botulinum toxin type B for migraine and tension headaches. Am. J. Health-Syst. Pharm..

[20] Grogan P.M., Alvarez M.V., Jones L. (2013). Headache direction and aura predict migraine responsiveness to rimabotulinumtoxin B. Headache.

[21] Lipton R.B., Varon S.F., Grosberg B., McAllister P.J., Freitag F., Aurora S.K., Dodick D.W., Silberstein S.D., Diener H.C., DeGryse R.E. (2011). Onabotulinumtoxina improves quality of life and reduces impact of chronic migraine. Neurology.

[22] Hollanda L., Monteiro L., Melo A. (2014). Botulinum toxin type A for cephalic cutaneous allodynia in chronic migraine: A randomized, double-blinded, placebo-controlled trial. Neurol. Int..

[23] Elkind A.H., O’Carroll P., Blumenfeld A., DeGryse R., Dimitrova R. (2006). A series of three sequential, randomized, controlled studies of repeated treatments with botulinum toxin type A for migraine prophylaxis. J. Pain.

[24] Relja M., Poole A.C., Schoenen J., Pascual J., Lei X., Thompson C. (2007). A multicentre, double-blind, randomized, placebo-controlled, parallel group study of multiple treatments of botulinum toxin type A (BoNTA) for the prophylaxis of episodic migraine headaches. Cephalalgia Int. J. Headache.

[25] Gady J., Ferneini E.M. (2013). Botulinum toxin A and headache treatment. Conn. Med..

[26] Peck M.W. (2009). Biology and genomic analysis of clostridium botulinum. Adv. Microb. Physiol..

[27] Stringer S.C., Carter A.T., Webb M.D., Wachnicka E., Crossman L.C., Sebaihia M., Peck M.W. (2013). Genomic and physiological variability within group II (non-proteolytic) clostridium botulinum. BMC genomics.

[28] Hill K.K., Smith T.J. (2013). Genetic diversity within clostridium botulinum serotypes, botulinum neurotoxin gene clusters and toxin subtypes. Curr. Top. Microbiol. Immunol..

[29] Gimenez D.F., Gimenez J.A. (1995). The typing of botulinal neurotoxins. Int. J. Food Microbiol..

[30] Montal M. (2010). Botulinum neurotoxin: A marvel of protein design. Ann. Rev. Biochem..

[31] Fischer A., Montal M. (2007). Single molecule detection of intermediates during botulinum neurotoxin translocation across membranes. Proc. Natl. Acad. Sci. USA.

[32] Fischer A., Nakai Y., Eubanks L.M., Clancy C.M., Tepp W.H., Pellett S., Dickerson T.J., Johnson E.A., Janda K.D., Montal M. (2009). Bimodal modulation of the botulinum neurotoxin protein-conducting channel. Proc. Natl. Acad. Sci. USA.

[33] Pirazzini M., Azarnia Tehran D., Zanetti G., Megighian A., Scorzeto M., Fillo S., Shone C.C., Binz T., Rossetto O., Lista F. (2014). Thioredoxin and its reductase are present on synaptic vesicles, and their inhibition prevents the paralysis induced by botulinum neurotoxins. Cell Rep..

[34] Montecucco C., Schiavo G. (1994). Mechanism of action of tetanus and botulinum neurotoxins. Mol. Microbiol..

[35] Whitemarsh R.C., Tepp W.H., Johnson E.A., Pellett S. (2014). Persistence of botulinum neurotoxin a subtypes 1–5 in primary rat spinal cord cells. PLoS ONE.

[36] Lacy D.B., Tepp W., Cohen A.C., DasGupta B.R., Stevens R.C. (1998). Crystal structure of botulinum neurotoxin type A and implications for toxicity. Nat. Struct. Biol..

[37] Mahowald M.L., Singh J.A., Dykstra D. (2006). Long term effects of intra-articular botulinum toxin A for refractory joint pain. Neurotox. Res..

[38] Castiglione A., Bagnato S., Boccagni C., Romano M.C., Galardi G. (2011). Efficacy of intra-articular injection of botulinum toxin type A in refractory hemiplegic shoulder pain. Arch. Phys. Med. Rehabil..

[39] Singh J.A., Fitzgerald P.M. (2011). Botulinum toxin for shoulder pain: A cochrane systematic review. J. Rheumatol..

[40] Boon A.J., Smith J., Dahm D.L., Sorenson E.J., Larson D.R., Fitz-Gibbon P.D., Dykstra D.D., Singh J.A. (2010). Efficacy of intra-articular botulinum toxin type A in painful knee osteoarthritis: A pilot study. PM & R J. Injury Funct. Rehabil..

[41] Ranoux D., Attal N., Morain F., Bouhassira D. (2008). Botulinum toxin type a induces direct analgesic effects in chronic neuropathic pain. Ann. Neurol..

[42] Fabregat G., Asensio-Samper J.M., Palmisani S., Villanueva-Perez V.L., de Andres J. (2013). Subcutaneous botulinum toxin for chronic post-thoracotomy pain. Pain Pract..

[43] Kitamura Y., Matsuka Y., Spigelman I., Ishihara Y., Yamamoto Y., Sonoyama W., Kamioka H., Yamashiro T., Kuboki T., Oguma K. (2009). Botulinum toxin type A (150 kda) decreases exaggerated neurotransmitter release from trigeminal ganglion neurons and relieves neuropathy behaviors induced by infraorbital nerve constriction. Neuroscience.

[44] Piovesan E.J., Teive H.G., Kowacs P.A., Della Coletta M.V., Werneck L.C., Silberstein S.D. (2005). An open study of botulinum-A toxin treatment of trigeminal neuralgia. Neurology.

[45] Zuniga C., Diaz S., Piedimonte F., Micheli F. (2008). Beneficial effects of botulinum toxin type A in trigeminal neuralgia. Arquivos Neuro-Psiquiatria.

[46] Turk U., Ilhan S., Alp R., Sur H. (2005). Botulinum toxin and intractable trigeminal neuralgia. Clin. Neuropharmacol..

[47] Wu C.J., Lian Y.J., Zheng Y.K., Zhang H.F., Chen Y., Xie N.C., Wang L.J. (2012). Botulinum toxin type A for the treatment of trigeminal neuralgia: Results from a randomized, double-blind, placebo-controlled trial. Cephalalgia Int. J. Headache.

[48] Guardiani E., Sadoughi B., Blitzer A., Sirois D. (2014). A new treatment paradigm for trigeminal neuralgia using botulinum toxin type A. Laryngoscope.

[49] Liu H.T., Tsai S.K., Kao M.C., Hu J.S. (2006). Botulinum toxin A relieved neuropathic pain in a case of post-herpetic neuralgia. Pain Med..

[50] Ruiz Huete C., Bermejo P. (2008). Botulinum toxin type A in the treatment of neuropathic pain in a case of postherpetic neuralgia. Neurologia.

[51] Xiao L., Mackey S., Hui H., Xong D., Zhang Q., Zhang D. (2010). Subcutaneous injection of botulinum toxin A is beneficial in postherpetic neuralgia. Pain Med..

[52] Yuan R.Y., Sheu J.J., Yu J.M., Chen W.T., Tseng I.J., Chang H.H., Hu C.J. (2009). Botulinum toxin for diabetic neuropathic pain: A randomized double-blind crossover trial. Neurology.

[53] Jabbari B. (2008). Evidence based medicine in the use of botulinum toxin for back pain. J. Neural Transm..

[54] Jabbari B. (2007). Treatment of chronic low back pain with botulinum neurotoxins. Curr. Pain Headache Rep..

[55] Cheshire W.P., Abashian S.W., Mann J.D. (1994). Botulinum toxin in the treatment of myofascial pain syndrome. Pain.

[56] Wheeler A.H., Goolkasian P., Gretz S.S. (1998). A randomized, double-blind, prospective pilot study of botulinum toxin injection for refractory, unilateral, cervicothoracic, paraspinal, myofascial pain syndrome. Spine.

[57] Gazerani P., Pedersen N.S., Staahl C., Drewes A.M., Arendt-Nielsen L. (2009). Subcutaneous botulinum toxin type A reduces capsaicin-induced trigeminal pain and vasomotor reactions in human skin. Pain.

[58] Gazerani P., Staahl C., Drewes A.M., Arendt-Nielsen L. (2006). The effects of botulinum toxin type A on capsaicin-evoked pain, flare, and secondary hyperalgesia in an experimental human model of trigeminal sensitization. Pain.

[59] Carmichael N.M., Dostrovsky J.O., Charlton M.P. (2010). Peptide-mediated transdermal delivery of botulinum neurotoxin type A reduces neurogenic inflammation in the skin. Pain.

[60] Tugnoli V., Capone J.G., Eleopra R., Quatrale R., Sensi M., Gastaldo E., Tola M.R., Geppetti P. (2007). Botulinum toxin type A reduces capsaicin-evoked pain and neurogenic vasodilatation in human skin. Pain.

[61] Schulte-Mattler W.J., Opatz O., Blersch W., May A., Bigalke H., Wohlfahrt K. (2007). Botulinum toxin A does not alter capsaicin-induced pain perception in human skin. J. Neurol. Sci..

[62] Blersch W., Schulte-Mattler W.J., Przywara S., May A., Bigalke H., Wohlfarth K. (2002). Botulinum toxin A and the cutaneous nociception in humans: A prospective, double-blind, placebo-controlled, randomized study. J. Neurol. Sci..

[63] Voller B., Sycha T., Gustorff B., Schmetterer L., Lehr S., Eichler H.G., Auff E., Schnider P. (2003). A randomized, double-blind, placebo controlled study on analgesic effects of botulinum toxin A. Neurology.

[64] Da Silva L.B., Kulas D., Karshenas A., Cairns B.E., Bach F.W., Arendt-Nielsen L., Gazerani P. (2014). Time course analysis of the effects of botulinum neurotoxin type A on pain and vasomotor responses evoked by glutamate injection into human temporalis muscles. Toxins.

[65] Sycha T., Samal D., Chizh B., Lehr S., Gustorff B., Schnider P., Auff E. (2006). A lack of antinociceptive or antiinflammatory effect of botulinum toxin A in an inflammatory human pain model. Anesth. Analg..

[66] Cui M., Khanijou S., Rubino J., Aoki K.R. (2004). Subcutaneous administration of botulinum toxin A reduces formalin-induced pain. Pain.

[67] Marino M.J., Terashima T., Steinauer J.J., Eddinger K.A., Yaksh T.L., Xu Q. (2014). Botulinum toxin B in the sensory afferent: Transmitter release, spinal activation, and pain behavior. Pain.

[68] Marinelli S., Luvisetto S., Cobianchi S., Makuch W., Obara I., Mezzaroma E., Caruso M., Straface E., Przewlocka B., Pavone F. (2010). Botulinum neurotoxin type A counteracts neuropathic pain and facilitates functional recovery after peripheral nerve injury in animal models. Neuroscience.

[69] Ma L., Nagai J., Sekino Y., Goto Y., Nakahira S., Ueda H. (2012). Single application of A2 NTX, a botulinum toxin A2 subunit, prevents chronic pain over long periods in both diabetic and spinal cord injury-induced neuropathic pain models. J. Pharmacol. Sci..

[70] Matak I., Lackovic Z. (2014). Botulinum toxin A, brain and pain. Prog. Neurobiol..

[71] Matak I., Bach-Rojecky L., Filipovic B., Lackovic Z. (2011). Behavioral and immunohistochemical evidence for central antinociceptive activity of botulinum toxin A. Neuroscience.

[72] Vacca V., Marinelli S., Eleuteri C., Luvisetto S., Pavone F. (2012). Botulinum neurotoxin A enhances the analgesic effects on inflammatory pain and antagonizes tolerance induced by morphine in mice. Brain Behav. Immun..

[73] Luvisetto S., Marinelli S., Lucchetti F., Marchi F., Cobianchi S., Rossetto O., Montecucco C., Pavone F. (2006). Botulinum neurotoxins and formalin-induced pain: Central *vs*. Peripheral effects in mice. Brain Res..

[74] Filipovic B., Matak I., Bach-Rojecky L., Lackovic Z. (2012). Central action of peripherally applied botulinum toxin type A on pain and dural protein extravasation in rat model of trigeminal neuropathy. PLoS ONE.

[75] Ramachandran R., Lam C., Yaksh T.L. (2015). Botulinum toxin in migraine: Role of transport in trigemino-somatic and trigemino-vascular afferents. Neurobiol. Dis..

[76] Bach-Rojecky L., Lackovic Z. (2005). Antinociceptive effect of botulinum toxin type A in rat model of carrageenan and capsaicin induced pain. Croat. Med. J..

[77] Bach-Rojecky L., Dominis M., Lackovic Z. (2008). Lack of anti-inflammatory effect of botulinum toxin type A in experimental models of inflammation. Fundam. Clin. Pharmacol..

[78] Favre-Guilmard C., Auguet M., Chabrier P.E. (2009). Different antinociceptive effects of botulinum toxin type A in inflammatory and peripheral polyneuropathic rat models. Eur. J. Pharmacol..

[79] Shin M.C., Yukihira T., Ito Y., Akaike N. (2013). Antinociceptive effects of A1 and A2 type botulinum toxins on carrageenan-induced hyperalgesia in rat. Toxicon.

[80] Krug H.E., Frizelle S., McGarraugh P., Mahowald M.L. (2009). Pain behavior measures to quantitate joint pain and response to neurotoxin treatment in murine models of arthritis. Pain Med..

[81] Heikkila H.M., Hielm-Bjorkman A.K., Morelius M., Larsen S., Honkavaara J., Innes J.F., Laitinen-Vapaavuori O.M. (2014). Intra-articular botulinum toxin A for the treatment of osteoarthritic joint pain in dogs: A randomized, double-blinded, placebo-controlled clinical trial. Vet. J..

[82] Hadley H.S., Wheeler J.L., Petersen S.W. (2010). Effects of intra-articular botulinum toxin type A (Botox^®^) in dogs with chronic osteoarthritis. Vet. Comp. Orthop. Traumatol..

[83] Rialland P., Authier S., Guillot M., del Castillo J.R., Veilleux-Lemieux D., Frank D., Gauvin D., Troncy E. (2012). Validation of orthopedic postoperative pain assessment methods for dogs: A prospective, blinded, randomized, placebo-controlled study. PLoS ONE.

[84] Park H.J., Marino M.J., Rondon E.S., Xu Q., Yaksh T.L. (2015). The effects of intraplantar and intrathecal botulinum toxin type B on tactile allodynia in mono and polyneuropathy in the mouse. Anesth. Analg..

[85] Park H.J., Lee Y., Lee J., Park C., Moon D.E. (2006). The effects of botulinum toxin A on mechanical and cold allodynia in a rat model of neuropathic pain. Can. J. Anaesth..

[86] Xiao L., Cheng J., Dai J., Zhang D. (2011). Botulinum toxin decreases hyperalgesia and inhibits P2X3 receptor over-expression in sensory neurons induced by ventral root transection in rats. Pain Med..

[87] Xiao L., Cheng J., Zhuang Y., Qu W., Muir J., Liang H., Zhang D. (2013). Botulinum toxin type A reduces hyperalgesia and trpv1 expression in rats with neuropathic pain. Pain Med..

[88] Bach-Rojecky L., Salkovic-Petrisic M., Lackovic Z. (2010). Botulinum toxin type A reduces pain supersensitivity in experimental diabetic neuropathy: Bilateral effect after unilateral injection. Eur. J. Pharmacol..

[89] Bach-Rojecky L., Lackovic Z. (2009). Central origin of the antinociceptive action of botulinum toxin type A. Pharmacol. Biochem. Behav..

[90] Shimizu T., Shibata M., Toriumi H., Iwashita T., Funakubo M., Sato H., Kuroi T., Ebine T., Koizumi K., Suzuki N. (2012). Reduction of trpv1 expression in the trigeminal system by botulinum neurotoxin type-A. Neurobiol. Dis..

[91] Gazerani P., Au S., Dong X., Kumar U., Arendt-Nielsen L., Cairns B.E. (2010). Botulinum neurotoxin type A (BoNTA) decreases the mechanical sensitivity of nociceptors and inhibits neurogenic vasodilation in a craniofacial muscle targeted for migraine prophylaxis. Pain.

[92] Meng J., Wang J., Lawrence G., Dolly J.O. (2007). Synaptobrevin i mediates exocytosis of cgrp from sensory neurons and inhibition by botulinum toxins reflects their anti-nociceptive potential. J. Cell Sci..

[93] Dolly J.O., O’Connell M.A. (2012). Neurotherapeutics to inhibit exocytosis from sensory neurons for the control of chronic pain. Curr. Opin. Pharmacol..

[94] Durham P.L., Cady R. (2004). Regulation of calcitonin gene-related peptide secretion from trigeminal nerve cells by botulinum toxin type A: Implications for migraine therapy. Headache.

[95] Edvinsson J., Warfvinge K., Edvinsson L. (2015). Modulation of inflammatory mediators in the trigeminal ganglion by botulinum neurotoxin type A: An organ culture study. J. Headache Pain.

[96] Silva L.B., Poulsen J.N., Arendt-Nielsen L., Gazerani P. (2015). Botulinum neurotoxin type a modulates vesicular release of glutamate from satellite glial cells. J. Cell Mol. Med..

[97] Willis W.D. (2007). The somatosensory system, with emphasis on structures important for pain. Brain Res. Rev..

[98] Bach-Rojecky L., Relja M., Lackovic Z. (2005). Botulinum toxin type A in experimental neuropathic pain. J. Neural Transm..

[99] Hoffman R.O., Helveston E.M. (1986). Botulinum in the treatment of adult motility disorders. Int. Ophthalmol. Clin..

[100] Ji R.R., Xu Z.Z., Gao Y.J. (2014). Emerging targets in neuroinflammation-driven chronic pain. Nat. Rev. Drug Discov..

[101] Yaksh T., Woller S., Ramachandran R., Sorkin L. (2015). The search for novel analgesics: Targets and mechanisms. F1000 Biol. Rep..

[102] Smith J.A., Davis C.L., Burgess G.M. (2000). Prostaglandin E2-induced sensitization of bradykinin-evoked responses in rat dorsal root ganglion neurons is mediated by cAMP-dependent protein kinase A. Eur. J. Neurosci..

[103] Ma W., Quirion R. (2007). Inflammatory mediators modulating the transient receptor potential vanilloid 1 receptor: Therapeutic targets to treat inflammatory and neuropathic pain. Expert Opin. Ther. Targets.

[104] Chahine M., O’Leary M.E. (2014). Regulation/modulation of sensory neuron sodium channels. Handb. Exp. Pharmacol..

[105] Gold M.S., Levine J.D., Correa A.M. (1998). Modulation of TTX-R ina by PKC and PKA and their role in PGE2-induced sensitization of rat sensory neurons *in vitro*. J. Neurosci. Off. J. Soc. Neurosci..

[106] Aley K.O., Levine J.D. (1999). Role of protein kinase A in the maintenance of inflammatory pain. J. Neurosci..

[107] Waxman S.G., Cummins T.R., Dib-Hajj S.D., Black J.A. (2000). Voltage-gated sodium channels and the molecular pathogenesis of pain: A review. J. Rehabil. Res. Dev..

[108] Suzuki R., Dickenson A. (2005). Spinal and supraspinal contributions to central sensitization in peripheral neuropathy. Neuro-Signals.

[109] Ji R.R., Kawasaki Y., Zhuang Z.Y., Wen Y.R., Zhang Y.Q. (2007). Protein kinases as potential targets for the treatment of pathological pain. Handb. Exp. Pharmacol..

[110] Crown E.D. (2012). The role of mitogen activated protein kinase signaling in microglia and neurons in the initiation and maintenance of chronic pain. Exp. Neurol..

[111] Edelmayer R.M., Brederson J.D., Jarvis M.F., Bitner R.S. (2014). Biochemical and pharmacological assessment of map-kinase signaling along pain pathways in experimental rodent models: A potential tool for the discovery of novel antinociceptive therapeutics. Biochem. Pharmacol..

[112] Choi J.I., Svensson C.I., Koehrn F.J., Bhuskute A., Sorkin L.S. (2010). Peripheral inflammation induces tumor necrosis factor dependent ampa receptor trafficking and akt phosphorylation in spinal cord in addition to pain behavior. Pain.

[113] Ma W., Quirion R. (2014). Targeting cell surface trafficking of pain-facilitating receptors to treat chronic pain conditions. Expert Opin. Ther. Targets.

[114] Kopach O., Krotov V., Belan P., Voitenko N. (2015). Inflammatory-induced changes in synaptic drive and postsynaptic ampars in lamina ii dorsal horn neurons are cell-type specific. Pain.

[115] Taves S., Berta T., Chen G., Ji R.R. (2013). Microglia and spinal cord synaptic plasticity in persistent pain. Neural Plast..

[116] Sofroniew M.V. (2014). Multiple roles for astrocytes as effectors of cytokines and inflammatory mediators. Neurosci. Rev. J. Bringing Neurobiol. Neurol. Psychiatry.

[117] Ramachandran R., Yaksh T.L. (2014). Therapeutic use of botulinum toxin in migraine: Mechanisms of action. Br. J. Pharmacol..

[118] Christianson C.A., Corr M., Firestein G.S., Mobargha A., Yaksh T.L., Svensson C.I. (2010). Characterization of the acute and persistent pain state present in K/BxN serum transfer arthritis. Pain.

[119] Bas D.B., Su J., Sandor K., Agalave N.M., Lundberg J., Codeluppi S., Baharpoor A., Nandakumar K.S., Holmdahl R., Svensson C.I. (2012). Collagen antibody-induced arthritis evokes persistent pain with spinal glial involvement and transient prostaglandin dependency. Arthritis Rheum..

[120] Jimenez-Andrade J.M., Mantyh P.W. (2012). Sensory and sympathetic nerve fibers undergo sprouting and neuroma formation in the painful arthritic joint of geriatric mice. Arthritis Res. Therapy.

[121] Xu Q., Yaksh T.L. (2011). A brief comparison of the pathophysiology of inflammatory versus neuropathic pain. Curr. Opin. Anaesthesiol..

[122] Taylor P., Manger B., Alvaro-Gracia J., Johnstone R., Gomez-Reino J., Eberhardt E., Wolfe F., Schwartzman S., Furfaro N., Kavanaugh A. (2010). Patient perceptions concerning pain management in the treatment of rheumatoid arthritis. J. Int. Med. Res..

[123] Wolfe F., Michaud K. (2007). Assessment of pain in rheumatoid arthritis: Minimal clinically significant difference, predictors, and the effect of anti-tumor necrosis factor therapy. J. Rheumatol..

[124] DePuy T., Howard R., Keegan K., Wilson D., Kramer J., Cook J.L., Childers M.K. (2007). Effects of intra-articular botulinum toxin type A in an equine model of acute synovitis: A pilot study. Am. J. Phys. Med. Rehabil./Assoc. Acad. Physiatr..

[125] Akaike N., Shin M.C., Wakita M., Torii Y., Harakawa T., Ginnaga A., Kato K., Kaji R., Kozaki S. (2013). Transsynaptic inhibition of spinal transmission by A2 botulinum toxin. J. Physiol..

[126] Mazzocchio R., Caleo M. (2015). More than at the neuromuscular synapse: Actions of botulinum neurotoxin A in the central nervous system. Neurosci. Rev. J. Bringing Neurobiol. Neurol. Psychiatry.

[127] Gerwin R. (2012). Botulinum toxin treatment of myofascial pain: A critical review of the literature. Curr. Pain Headache Rep..

[128] Avendano-Coy J., Gomez-Soriano J., Valencia M., Estrada J., Leal F., Ruiz-Campa R. (2014). Botulinum toxin type A and myofascial pain syndrome: A retrospective study of 301 patients. J. Back Musculoskelet. Rehabil..

[129] Zhou J.Y., Wang D. (2014). An update on botulinum toxin A injections of trigger points for myofascial pain. Curr. Pain Headache Rep..

[130] Desai M.J., Shkolnikova T., Nava A., Inwald D. (2014). A critical appraisal of the evidence for botulinum toxin type A in the treatment for cervico-thoracic myofascial pain syndrome. Pain Pract. Off. J. World Inst. Pain.

[131] Singh J.A., Mahowald M.L., Kushnaryov A., Goelz E., Dykstra D. (2009). Repeat injections of intra-articular botulinum toxin A for the treatment of chronic arthritis joint pain. J. Clin. Rheumatol. Pract. Rep. Rheum. Musculoskelet. Dis..

[132] Kramer H.H., Angerer C., Erbguth F., Schmelz M., Birklein F. (2003). Botulinum toxin A reduces neurogenic flare but has almost no effect on pain and hyperalgesia in human skin. J. Neurol..

[133] Sorkin L.S., Yaksh T.L. (2009). Behavioral models of pain states evoked by physical injury to the peripheral nerve. NeuroTherapeutics.

[134] Challa S.R. (2015). Surgical animal models of neuropathic pain: Pros and Cons. Int. J. Neurosci..

[135] Siau C., Xiao W., Bennett G.J. (2006). Paclitaxel- and vincristine-evoked painful peripheral neuropathies: Loss of epidermal innervation and activation of langerhans cells. Exp. Neurol..

[136] Tesch G.H., Allen T.J. (2007). Rodent models of streptozotocin-induced diabetic nephropathy. Nephrology.

[137] Devor M. (2006). Sodium channels and mechanisms of neuropathic pain. J. Pain..

[138] Amir R., Devor M. (2003). Extra spike formation in sensory neurons and the disruption of afferent spike patterning. Biophys. J..

[139] Tsujino H., Kondo E., Fukuoka T., Dai Y., Tokunaga A., Miki K., Yonenobu K., Ochi T., Noguchi K. (2000). Activating transcription factor 3 (ATF3) induction by axotomy in sensory and motoneurons: A novel neuronal marker of nerve injury. Mol. Cell. Neurosci..

[140] Strickland I.T., Martindale J.C., Woodhams P.L., Reeve A.J., Chessell I.P., McQueen D.S. (2008). Changes in the expression of NaV1.7, NaV1.8 and NaV1.9 in a distinct population of dorsal root ganglia innervating the rat knee joint in a model of chronic inflammatory joint pain. Eur. J. Pain.

[141] Chien L.Y., Cheng J.K., Chu D., Cheng C.F., Tsaur M.L. (2007). Reduced expression of a-type potassium channels in primary sensory neurons induces mechanical hypersensitivity. J. Neurosci..

[142] Burnstock G., Krugel U., Abbracchio M.P., Illes P. (2011). Purinergic signalling: From normal behaviour to pathological brain function. Prog. Neurobiol..

[143] Chang Y.W., Winkelstein B.A. (2011). Schwann cell proliferation and macrophage infiltration are evident at day 14 after painful cervical nerve root compression in the rat. J. Neurotrauma.

[144] Abbadie C., Lindia J.A., Cumiskey A.M., Peterson L.B., Mudgett J.S., Bayne E.K., DeMartino J.A., MacIntyre D.E., Forrest M.J. (2003). Impaired neuropathic pain responses in mice lacking the chemokine receptor CCR2. Proc. Natl. Acad Sci. USA.

[145] Tofaris G.K., Patterson P.H., Jessen K.R., Mirsky R. (2002). Denervated schwann cells attract macrophages by secretion of leukemia inhibitory factor (LIF) and monocyte chemoattractant protein-1 in a process regulated by interleukin-6 and LIF. J. Neurosci..

[146] McLachlan E.M., Hu P. (2014). Inflammation in dorsal root ganglia after peripheral nerve injury: Effects of the sympathetic innervation. Auton. Neurosci. Basic Clin..

[147] Blum E., Procacci P., Conte V., Hanani M. (2014). Systemic inflammation alters satellite glial cell function and structure. A possible contribution to pain. Neuroscience.

[148] Ma C., Shu Y., Zheng Z., Chen Y., Yao H., Greenquist K.W., White F.A., LaMotte R.H. (2003). Similar electrophysiological changes in axotomized and neighboring intact dorsal root ganglion neurons. J. Neurophysiol..

[149] Huang L.Y., Gu Y., Chen Y. (2013). Communication between neuronal somata and satellite glial cells in sensory ganglia. Glia.

[150] Sukhotinsky I., Ben-Dor E., Raber P., Devor M. (2004). Key role of the dorsal root ganglion in neuropathic tactile hypersensibility. Eur. J. Pain.

[151] Ellis A., Bennett D.L. (2013). Neuroinflammation and the generation of neuropathic pain. Br. J. Anaesth..

[152] Ji R.R., Berta T., Nedergaard M. (2013). Glia and pain: Is chronic pain a gliopathy?. Pain.

[153] Gobel H., Heinze A., Heinze-Kuhn K., Jost W.H. (2001). Evidence-based medicine: Botulinum toxin A in migraine and tension-type headache. J. Neurol..

[154] Wheeler A., Smith H.S. (2013). Botulinum toxins: Mechanisms of action, antinociception and clinical applications. Toxicology.

[155] Quick M.W. (2006). The role of snare proteins in trafficking and function of neurotransmitter transporters. Handb. Exp. Pharmacol..

[156] Schrattenholz A., Soskic V. (2006). Nmda receptors are not alone: Dynamic regulation of nmda receptor structure and function by neuregulins and transient cholesterol-rich membrane domains leads to disease-specific nuances of glutamate-signalling. Curr. Top. Med. Chem..

[157] Hou Q., Huang Y., Amato S., Snyder S.H., Huganir R.L., Man H.Y. (2008). Regulation of ampa receptor localization in lipid rafts. Mol. Cell. Neurosci..

[158] Patel H.H., Murray F., Insel P.A. (2008). G-protein-coupled receptor-signaling components in membrane raft and caveolae microdomains. Handb. Exp. Pharmacol..

[159] Delint-Ramirez I., Fernandez E., Bayes A., Kicsi E., Komiyama N.H., Grant S.G. (2010). *In vivo* composition of nmda receptor signaling complexes differs between membrane subdomains and is modulated by PSD-95 and PSD-93. J. Neurosci..

[160] Fessler M.B., Parks J.S. (2011). Intracellular lipid flux and membrane microdomains as organizing principles in inflammatory cell signaling. J. Immunol..

[161] Kakegawa W., Yuzaki M. (2005). A mechanism underlying ampa receptor trafficking during cerebellar long-term potentiation. Proc. Natl. Acad. Sci. USA.

[162] Verderio C., Pozzi D., Pravettoni E., Inverardi F., Schenk U., Coco S., Proux-Gillardeaux V., Galli T., Rossetto O., Frassoni C. (2004). Snap-25 modulation of calcium dynamics underlies differences in gabaergic and glutamatergic responsiveness to depolarization. Neuron.

[163] Pozzi D., Condliffe S., Bozzi Y., Chikhladze M., Grumelli C., Proux-Gillardeaux V., Takahashi M., Franceschetti S., Verderio C., Matteoli M. (2008). Activity-dependent phosphorylation of SER187 is required for snap-25-negative modulation of neuronal voltage-gated calcium channels. Proc. Natl. Acad. Sci. USA.

[164] Gerachshenko T., Blackmer T., Yoon E.J., Bartleson C., Hamm H.E., Alford S. (2005). Gbetagamma acts at the C terminus of SNAP-25 to mediate presynaptic inhibition. Nat. Neurosci..

[165] Morenilla-Palao C., Planells-Cases R., García-Sanz N., Ferrer-Montiel A. (2004). Regulated exocytosis contributes to protein kinase C potentiation of vanilloid receptor activity. J. Biol. Chem..

[166] Pellett S. (2013). Progress in cell based assays for botulinum neurotoxin detection. Curr. Top. Microbiol. Immunol..

[167] Richardson J.D., Vasko M.R. (2002). Cellular mechanisms of neurogenic inflammation. J. Pharmacol. Exp. Ther..

[168] Woolf C.J., Ma Q. (2007). Nociceptors-noxious stimulus detectors. Neuron.

[169] Hucho T., Levine J.D. (2007). Signaling pathways in sensitization: Toward a nociceptor cell biology. Neuron.

[170] Ernst J.D. (2000). Bacterial inhibition of phagocytosis. Cell. Microbiol..

[171] Mollinedo F., Calafat J., Janssen H., Martin-Martin B., Canchado J., Nabokina S.M., Gajate C. (2006). Combinatorial snare complexes modulate the secretion of cytoplasmic granules in human neutrophils. J. Immunol..

[172] Murray R.Z., Stow J.L. (2014). Cytokine secretion in macrophages: Snares, rabs, and membrane trafficking. Front. Immunol..

[173] Tecchio C., Micheletti A., Cassatella M.A. (2014). Neutrophil-derived cytokines: Facts beyond expression. Front. Immunol..

[174] Sheshachalam A., Srivastava N., Mitchell T., Lacy P., Eitzen G. (2014). Granule protein processing and regulated secretion in neutrophils. Front. Immunol..

[175] Moon T.C., Befus A.D., Kulka M. (2014). Mast cell mediators: Their differential release and the secretory pathways involved. Front. Immunol..

[176] Logan M.R., Odemuyiwa S.O., Moqbel R. (2003). Understanding exocytosis in immune and inflammatory cells: The molecular basis of mediator secretion. J. Allergyd Clin. Immunol..

[177] Lorentz A., Baumann A., Vitte J., Blank U. (2012). The snare machinery in mast cell secretion. Front. Immunol..

[178] Park T.H. (2013). The effects of botulinum toxin A on mast cell activity: Preliminary results. Burns J. Int. Soc. Burn Inj..

[179] Akhtar N., Brooks P. (2012). The use of botulinum toxin in the management of burns itching: Preliminary results. Burns.

[180] Burstein R., Yamamura H., Malick A., Strassman A.M. (1998). Chemical stimulation of the intracranial dura induces enhanced responses to facial stimulation in brain stem trigeminal neurons. J. Neurophysiol..

[181] Burstein R., Zhang X., Levy D., Aoki K.R., Brin M.F. (2014). Selective inhibition of meningeal nociceptors by botulinum neurotoxin type A: Therapeutic implications for migraine and other pains. Cephalalgia Int. J. Headache.

[182] Salaun C., Gould G.W., Chamberlain L.H. (2005). Lipid raft association of snare proteins regulates exocytosis in PC12 cells. J. Biol. Chem..

[183] Owen D.M., Magenau A., Williamson D., Gaus K. (2012). The lipid raft hypothesis revisited—New insights on raft composition and function from super-resolution fluorescence microscopy. BioEssays.

[184] Meng J., Ovsepian S.V., Wang J., Pickering M., Sasse A., Aoki K.R., Lawrence G.W., Dolly J.O. (2009). Activation of trpv1 mediates calcitonin gene-related peptide release, which excites trigeminal sensory neurons and is attenuated by a retargeted botulinum toxin with anti-nociceptive potential. J. Neurosci..

[185] Montell C. (2004). Exciting trips for TRPS. Nat. Cell Biol..

[186] Lan J.Y., Skeberdis V.A., Jover T., Grooms S.Y., Lin Y., Araneda R.C., Zheng X., Bennett M.V., Zukin R.S. (2001). Protein kinase C modulates nmda receptor trafficking and gating. Nat. Neurosci..

[187] Planells-Cases R., Ferrer-Montiel A., Liedtke W.B., Heller S. (2007). TRP Channel Trafficking. TRP Ion Channel Function in Sensory Transduction and Cellular Signaling Cascades.

[188] Ferrandiz-Huertas C., Mathivanan S., Wolf C.J., Devesa I., Ferrer-Montiel A. (2014). Trafficking of thermotrp channels. Membranes.

[189] Yaksh T.L., Ozaki G., McCumber D., Rathbun M., Svensson C., Malkmus S., Yaksh M.C. (2001). An automated flinch detecting system for use in the formalin nociceptive bioassay. J. Appl. Physiol..

[190] Antonucci F., Rossi C., Gianfranceschi L., Rossetto O., Caleo M. (2008). Long-distance retrograde effects of botulinum neurotoxin A. J. Neurosci..

[191] Restani L., Antonucci F., Gianfranceschi L., Rossi C., Rossetto O., Caleo M. (2011). Evidence for anterograde transport and transcytosis of botulinum neurotoxin A (BoNT/A). J. Neurosci..

[192] Restani L., Giribaldi F., Manich M., Bercsenyi K., Menendez G., Rossetto O., Caleo M., Schiavo G. (2012). Botulinum neurotoxins A and e undergo retrograde axonal transport in primary motor neurons. PLoS Pathog..

[193] Lawrence G.W., Ovsepian S.V., Wang J., Aoki K.R., Dolly J.O. (2012). Extravesicular intraneuronal migration of internalized botulinum neurotoxins without detectable inhibition of distal neurotransmission. Biochem. J..

[194] Habermann E. (1974). 125I-labeled neurotoxin from clostridium botulinum a: Preparation, binding to synaptosomes and ascent to the spinal cord. Naunyn-Schmiedeberg’s Arch. Pharmacol..

[195] Wiegand H., Erdmann G., Wellhoner H.H. (1976). 125I-labelled botulinum A neurotoxin: Pharmacokinetics in cats after intramuscular injection. Naunyn-Schmiedeberg's Arch. Pharmacol..

[196] Black J.D., Dolly J.O. (1986). Interaction of 125I-labeled botulinum neurotoxins with nerve terminals. I. Ultrastructural autoradiographic localization and quantitation of distinct membrane acceptors for types A and B on motor nerves. J. Cell Biol..

[197] Matak I., Riederer P., Lackovic Z. (2012). Botulinum toxin’s axonal transport from periphery to the spinal cord. Neurochem. Int..

[198] Aoki K.R. (2003). Evidence for antinociceptive activity of botulinum toxin type A in pain management. Headache.

[199] Koizumi H., Goto S., Okita S., Morigaki R., Akaike N., Torii Y., Harakawa T., Ginnaga A., Kaji R. (2014). Spinal central effects of peripherally applied botulinum neurotoxin A in comparison between its subtypes A1 and A2. Front. Neurol..

[200] Restani L., Novelli E., Bottari D., Leone P., Barone I., Galli-Resta L., Strettoi E., Caleo M. (2012). Botulinum neurotoxin A impairs neurotransmission following retrograde transynaptic transport. Traffic.

[201] Alexiades-Armenakas M. (2008). Retrograde transport and transcytosis of botulinum toxin serotypes to the brain: Analysis of potential neurotoxicity. J. Drugs Dermatol. JDD.

[202] Marchand-Pauvert V., Aymard C., Giboin L.S., Dominici F., Rossi A., Mazzocchio R. (2013). Beyond muscular effects: Depression of spinal recurrent inhibition after botulinum neurotoxin A. J. Physiol..

[203] Takasusuki T., Yaksh T.L. (2011). The effects of intrathecal and systemic gabapentin on spinal substance p release. Anesth. Analg..

[204] Yaksh T.L. (2006). Calcium channels as therapeutic targets in neuropathic pain. J. Pain.

[205] Malmberg A.B., Yaksh T.L. (1995). Effect of continuous intrathecal infusion of omega-conopeptides, n-type calcium-channel blockers, on behavior and antinociception in the formalin and hot-plate tests in rats. Pain.

[206] Adams D.J., Callaghan B., Berecki G. (2012). Analgesic conotoxins: Block and G protein-coupled receptor modulation of N-type (CaV 2.2) calcium channels. Br. J. Pharmacol..

[207] Terashima T., Xu Q., Yamaguchi S., Yaksh T.L. (2013). Intrathecal P/Q- and R-type calcium channel blockade of spinal substance P release and c-Fos expression. Neuropharmacology.

[208] Chaplan S.R., Bach F.W., Pogrel J.W., Chung J.M., Yaksh T.L. (1994). Quantitative assessment of tactile allodynia in the rat paw. J. Neurosci. Methods.

[209] Chaplan S.R., Pogrel J.W., Yaksh T.L. (1994). Role of voltage-dependent calcium channel subtypes in experimental tactile allodynia. J. Pharmacol. Exp. Ther..

[210] Xiao Y., Richter J.A., Hurley J.H. (2008). Release of glutamate and cgrp from trigeminal ganglion neurons: Role of calcium channels and 5-ht1 receptor signaling. Mol. Pain.

[211] Gu J.G., MacDermott A.B. (1997). Activation of ATP P2X receptors elicits glutamate release from sensory neuron synapses. Nature.

[212] Nguyen D., Deng P., Matthews E.A., Kim D.S., Feng G., Dickenson A.H., Xu Z.C., Luo Z.D. (2009). Enhanced pre-synaptic glutamate release in deep-dorsal horn contributes to calcium channel alpha-2-delta-1 protein-mediated spinal sensitization and behavioral hypersensitivity. Mol. Pain.

[213] Shinder V., Amir R., Devor M. (1998). Cross-excitation in dorsal root ganglia does not depend on close cell-to-cell apposition. Neuroreport.

[214] Devor M., Wall P.D. (1990). Cross-excitation in dorsal root ganglia of nerve-injured and intact rats. J. Neurophysiol..

[215] Amir R., Devor M. (2000). Functional cross-excitation between afferent a- and c-neurons in dorsal root ganglia. Neuroscience.

[216] Kung L.H., Gong K., Adedoyin M., Ng J., Bhargava A., Ohara P.T., Jasmin L. (2013). Evidence for glutamate as a neuroglial transmitter within sensory ganglia. PLoS ONE.

[217] Ferrari L.F., Lotufo C.M., Araldi D., Rodrigues M.A., Macedo L.P., Ferreira S.H., Parada C.A. (2014). Inflammatory sensitization of nociceptors depends on activation of nmda receptors in drg satellite cells. Proc. Natl. Acad. Sci. USA.

[218] Omoto K., Maruhama K., Terayama R., Yamamoto Y., Matsushita O., Sugimoto T., Oguma K., Matsuka Y. (2015). Cross-excitation in peripheral sensory ganglia associated with pain transmission. Toxins.

[219] Steinberg J.P., Huganir R.L., Linden D.J. (2004). N-ethylmaleimide-sensitive factor is required for the synaptic incorporation and removal of ampa receptors during cerebellar long-term depression. Proc. Natl. Acad. Sci. USA.

[220] Lau C.G., Takayasu Y., Rodenas-Ruano A., Paternain A.V., Lerma J., Bennett M.V., Zukin R.S. (2010). SNAP-25 is a target of protein kinase C phosphorylation critical to NMDA receptor trafficking. J. Neurosci..

[221] Larsson M., Broman J. (2011). Synaptic plasticity and pain: Role of ionotropic glutamate receptors. Neuroscientist.

[222] Bardoni R. (2013). Role of presynaptic glutamate receptors in pain transmission at the spinal cord level. Curr. Neuropharmacol..

[223] Walker S.M., Beggs S., Baccei M.L. (2015). Persistent changes in peripheral and spinal nociceptive processing after early tissue injury. Exp. Neurol..

[224] Huang P.P., Khan I., Suhail M.S., Malkmus S., Yaksh T.L. (2011). Spinal botulinum neurotoxin B: Effects on afferent transmitter release and nociceptive processing. PLoS ONE.

[225] Lee W.H., Shin T.J., Kim H.J., Lee J.K., Suh H.W., Lee S.C., Seo K. (2011). Intrathecal administration of botulinum neurotoxin type A attenuates formalin-induced nociceptive responses in mice. Anesth. Analg..

[226] Coelho A., Oliveira R., Rossetto O., Cruz C.D., Cruz F., Avelino A. (2014). Intrathecal administration of botulinum toxin type A improves urinary bladder function and reduces pain in rats with cystitis. Eur. J. Pain.

[227] Schiavo G., Benfenati F., Poulain B., Rossetto O., Polverino de Laureto P., DasGupta B.R., Montecucco C. (1992). Tetanus and botulinum-B neurotoxins block neurotransmitter release by proteolytic cleavage of synaptobrevin. Nature.

[228] Verderio C., Grumelli C., Raiteri L., Coco S., Paluzzi S., Caccin P., Rossetto O., Bonanno G., Montecucco C., Matteoli M. (2007). Traffic of botulinum toxins A and e in excitatory and inhibitory neurons. Traffic.

[229] Dolly J.O., Lawrence G.W., Meng J., Wang J., Ovsepian S.V. (2009). Neuro-exocytosis: Botulinum toxins as inhibitory probes and versatile therapeutics. Curr. Opin. Pharmacol..

[230] Habermann E. (1988). Inhibition by tetanus and botulinum A toxin of the release of [3H]noradrenaline and [3H]gaba from rat brain homogenate. Experientia.

[231] Carroll I., Fischbein N., Barad M., Mackey S. (2011). Human response to unintended intrathecal injection of botulinum toxin. Pain Med..

[232] Marinelli S., Vacca V., Ricordy R., Uggenti C., Tata A.M., Luvisetto S., Pavone F. (2012). The analgesic effect on neuropathic pain of retrogradely transported botulinum neurotoxin a involves schwann cells and astrocytes. PLoS ONE.

[233] Littlewood N.K., Todd A.J., Spike R.C., Watt C., Shehab S.A. (1995). The types of neuron in spinal dorsal horn which possess neurokinin-1 receptors. Neuroscience.

[234] Scholz J., Woolf C.J. (2007). The neuropathic pain triad: Neurons, immune cells and glia. Nat. Neurosci..

[235] Peng H., Kang N., Xu J., Stanton P.K., Kang J. (2013). Two distinct modes of exocytotic fusion pore expansion in large astrocytic vesicles. J. Biol. Chem..

[236] Verderio C., Cagnoli C., Bergami M., Francolini M., Schenk U., Colombo A., Riganti L., Frassoni C., Zuccaro E., Danglot L. (2012). Ti-VAMP/VAMP7 is the snare of secretory lysosomes contributing to ATP secretion from astrocytes. Biol. Cell.

[237] Prada I., Marchaland J., Podini P., Magrassi L., D'Alessandro R., Bezzi P., Meldolesi J. (2011). REST/NRSF governs the expression of dense-core vesicle gliosecretion in astrocytes. J. Cell Biol..

[238] Hepp R., Perraut M., Chasserot-Golaz S., Galli T., Aunis D., Langley K., Grant N.J. (1999). Cultured glial cells express the SNAP-25 analogue SNAP-23. Glia.

[239] Parpura V., Fang Y., Basarsky T., Jahn R., Haydon P.G. (1995). Expression of synaptobrevin II, cellubrevin and syntaxin but not SNAP-25 in cultured astrocytes. FEBS Lett..

[240] Schubert V., Bouvier D., Volterra A. (2011). Snare protein expression in synaptic terminals and astrocytes in the adult hippocampus: A comparative analysis. Glia.

[241] Jeftinija S.D., Jeftinija K.V., Stefanovic G. (1997). Cultured astrocytes express proteins involved in vesicular glutamate release. Brain Res..

[242] Maienschein V., Marxen M., Volknandt W., Zimmermann H. (1999). A plethora of presynaptic proteins associated with ATP-storing organelles in cultured astrocytes. Glia.

[243] Kim H.J., Lee G.W., Kim M.J., Yang K.Y., Kim S.T., Bae Y.C., Ahn D.K. (2015). Antinociceptive effects of transcytosed botulinum neurotoxin type A on trigeminal nociception in rats. Korean J. Physiol. Pharmacol..

[244] Dover N., Barash J.R., Hill K.K., Xie G., Arnon S.S. (2014). Molecular characterization of a novel botulinum neurotoxin type H gene. J. Infect. Dis..

[245] Barash J.R., Arnon S.S. (2014). A novel strain of clostridium botulinum that produces type B and type H botulinum toxins. J. Infect. Dis..

[246] Kalb S.R., Baudys J., Raphael B.H., Dykes J.K., Luquez C., Maslanka S.E., Barr J.R. (2015). Functional characterization of botulinum neurotoxin serotype H as a hybrid of known serotypes F and A (BoNT F/A). Anal. Chem..

[247] Kalb S.R., Baudys J., Webb R.P., Wright P., Smith T.J., Smith L.A., Fernandez R., Raphael B.H., Maslanka S.E., Pirkle J.L. (2012). Discovery of a novel enzymatic cleavage site for botulinum neurotoxin F5. FEBS Lett..

[248] Rummel A. (2013). Double receptor anchorage of botulinum neurotoxins accounts for their exquisite neurospecificity. Curr. Top. Microbiol. Immunol..

[249] Dong M., Yeh F., Tepp W.H., Dean C., Johnson E.A., Janz R., Chapman E.R. (2006). Sv2 is the protein receptor for botulinum neurotoxin a. Science.

[250] Jacky B.P., Garay P.E., Dupuy J., Nelson J.B., Cai B., Molina Y., Wang J., Steward L.E., Broide R.S., Francis J. (2013). Identification of fibroblast growth factor receptor 3 (FGFR3) as a protein receptor for botulinum neurotoxin serotype A (BoNT/A). PLoS Pathog..

[251] Grothe C., Meisinger C., Claus P. (2001). *In vivo* expression and localization of the fibroblast growth factor system in the intact and lesioned rat peripheral nerve and spinal ganglia. J. Comp. Neurol..

[252] Jungnickel J., Gransalke K., Timmer M., Grothe C. (2004). Fibroblast growth factor receptor 3 signaling regulates injury-related effects in the peripheral nervous system. Mol. Cell. Neurosci..

[253] Pringle N.P., Yu W.P., Howell M., Colvin J.S., Ornitz D.M., Richardson W.D. (2003). FGFR3 expression by astrocytes and their precursors: Evidence that astrocytes and oligodendrocytes originate in distinct neuroepithelial domains. Development.

[254] Niidome T., Nonaka H., Akaike A., Kihara T., Sugimoto H. (2009). Basic fibroblast growth factor promotes the generation of microtubule-associated protein 2-positive cells from microglia. Biochem. Biophys. Res. Commun..

[255] Peng L., Adler M., Demogines A., Borrell A., Liu H., Tao L., Tepp W.H., Zhang S.C., Johnson E.A., Sawyer S.L. (2014). Widespread sequence variations in vamp1 across vertebrates suggest a potential selective pressure from botulinum neurotoxins. PLoS Pathog..

[256] Yamasaki S., Baumeister A., Binz T., Blasi J., Link E., Cornille F., Roques B., Fykse E.M., Sudhof T.C., Jahn R. (1994). Cleavage of members of the synaptobrevin/VAMP family by types D and F botulinal neurotoxins and tetanus toxin. J. Biol. Chem..

[257] Yamamoto H., Ida T., Tsutsuki H., Mori M., Matsumoto T., Kohda T., Mukamoto M., Goshima N., Kozaki S., Ihara H. (2012). Specificity of botulinum protease for human vamp family proteins. Microbiol. Immunol..

[258] Strotmeier J., Willjes G., Binz T., Rummel A. (2012). Human synaptotagmin-ii is not a high affinity receptor for botulinum neurotoxin B and G: Increased therapeutic dosage and immunogenicity. FEBS Lett..

[259] Peng L., Berntsson R.P., Tepp W.H., Pitkin R.M., Johnson E.A., Stenmark P., Dong M. (2012). Botulinum neurotoxin D-C uses synaptotagmin I/II as receptors and human synaptotagmin II is not an effective receptor for type B, D-C, and G toxins. J. Cell Sci..

[260] Johnson E.A., Borriello S.P., Murray P.R., Funke G. (2005). Clostridium botulinum and clostridium tetani. Topley and Wilson’s Microbiology and Microbial Infections.

[261] Eleopra R., Tugnoli V., Quatrale R., Rossetto O., Montecucco C. (2004). Different types of botulinum toxin in humans. Mov. Disord..

[262] Sloop R.R., Cole B.A., Escutin R.O. (1997). Human response to botulinum toxin injection: Type B compared with type A. Neurology.

[263] Foran P.G., Mohammed N., Lisk G.O., Nagwaney S., Lawrence G.W., Johnson E., Smith L., Aoki K.R., Dolly J.O. (2003). Evaluation of the therapeutic usefulness of botulinum neurotoxin B, C1, E, and F compared with the long lasting type A. Basis for distinct durations of inhibition of exocytosis in central neurons. J. Biol. Chem..

[264] Keller J.E. (2006). Recovery from botulinum neurotoxin poisoning *in vivo*. Neuroscience.

[265] Eleopra R., Tugnoli V., Quatrale R., Rossetto O., Montecucco C., Dressler D. (2006). Clinical use of non-A botulinum toxins: Botulinum toxin type c and botulinum toxin type F. Neurotox. Res..

[266] Eleopra R., Tugnoli V., Rossetto O., Montecucco C., de Grandis D. (1997). Botulinum neurotoxin serotype C: A novel effective botulinum toxin therapy in human. Neurosci. Lett..

[267] Houser M.K., Sheean G.L., Lees A.J. (1998). Further studies using higher doses of botulinum toxin type F for torticollis resistant to botulinum toxin type A. J. Neurol. Neurosurg. Psychiatry.

[268] Sheean G.L., Lees A.J. (1995). Botulinum toxin F in the treatment of torticollis clinically resistant to botulinum toxin A. J. Neurol. Neurosurg. Psychiatry.

[269] Greene P.E., Fahn S. (1993). Use of botulinum toxin type F injections to treat torticollis in patients with immunity to botulinum toxin type A. Mov. Disord..

[270] Eleopra R., Montecucco C., Devigili G., Lettieri C., Rinaldo S., Verriello L., Pirazzini M., Caccin P., Rossetto O. (2013). Botulinum neurotoxin serotype D is poorly effective in humans: An *in vivo* electrophysiological study. Clin. Neurophysiol..

[271] Pellett S., Tepp W.H., Scherf J.M., Pier C.L., Johnson E.A. (2015). Activity of botulinum neurotoxin type d (strain 1873) in human neurons. Toxicon.

[272] Whitemarsh R.C., Tepp W.H., Bradshaw M., Lin G., Pier C.L., Scherf J.M., Johnson E.A., Pellett S. (2013). Characterization of botulinum neurotoxin a subtypes 1 through 5 by investigation of activities in mice, neuronal cell cultures, and *in vitro*. Infect. Immun..

[273] Wang D., Krilich J., Pellett S., Baudys J., Tepp W.H., Barr J.R., Johnson E.A., Kalb S.R. (2013). Comparison of the catalytic properties of the botulinum neurotoxin subtypes A1 and A5. Biochim. Biophys. Acta.

[274] Pier C.L., Chen C., Tepp W.H., Lin G., Janda K.D., Barbieri J.T., Pellett S., Johnson E.A. (2011). Botulinum neurotoxin subtype A2 enters neuronal cells faster than subtype a1. FEBS Lett..

[275] Tepp W.H., Lin G., Johnson E.A. (2012). Purification and characterization of a novel subtype A3 botulinum neurotoxin. Appl. Environ. Microbiol..

[276] Kull S., Schulz K.M., Weisemann J., Kirchner S., Schreiber T., Bollenbach A., Dabrowski P.W., Nitsche A., Kalb S.R., Dorner M.B. (2015). Isolation and functional characterization of the novel clostridium botulinum neurotoxin A8 subtype. PLoS ONE.

[277] Pellett S., Tepp W.H., Whitemarsh R.C., Bradshaw M., Johnson E.A. (2015). *In vivo* onset and duration of action varies for botulinum neurotoxin A subtypes 1–5. Toxicon.

[278] Torii Y., Goto Y., Nakahira S., Kozaki S., Kaji R., Ginnaga A. (2014). Comparison of systemic toxicity between botulinum toxin subtypes A1 and A2 in mice and rats. Basic Clin. Pharmacol. Toxicol..

[279] Torii Y., Akaike N., Harakawa T., Kato K., Sugimoto N., Goto Y., Nakahira S., Kohda T., Kozaki S., Kaji R. (2011). Type A1 but not type A2 botulinum toxin decreases the grip strength of the contralateral foreleg through axonal transport from the toxin-treated foreleg of rats. J. Pharmacol. Sci..

[280] Torii Y., Kiyota N., Sugimoto N., Mori Y., Goto Y., Harakawa T., Nakahira S., Kaji R., Kozaki S., Ginnaga A. (2010). Comparison of effects of botulinum toxin subtype A1 and A2 using twitch tension assay and rat grip strength test. Toxicon Off. J. Int. Soc. Toxinol..

[281] Mukai Y., Shimatani Y., Sako W., Asanuma K., Nodera H., Sakamoto T., Izumi Y., Kohda T., Kozaki S., Kaji R. (2014). Comparison between botulinum neurotoxin type A2 and type A1 by electrophysiological study in healthy individuals. Toxicon.

[282] Lin G., Tepp W.H., Pier C.L., Jacobson M.J., Johnson E.A. (2010). Expression of the clostridium botulinum A2 neurotoxin gene cluster proteins and characterization of the A2 complex. Appl. Environ. Microbiol..

[283] Akaike N., Ito Y., Shin M.C., Nonaka K., Torii Y., Harakawa T., Ginnaga A., Kozaki S., Kaji R. (2010). Effects of A2 type botulinum toxin on spontaneous miniature and evoked transmitter release from the rat spinal excitatory and inhibitory synapses. Toxicon.

[284] Henkel J.S., Jacobson M., Tepp W., Pier C., Johnson E.A., Barbieri J.T. (2009). Catalytic properties of botulinum neurotoxin subtypes A3 and A4 (dagger). Biochemistry.

[285] Wang J., Meng J., Lawrence G.W., Zurawski T.H., Sasse A., Bodeker M.O., Gilmore M.A., Fernandez-Salas E., Francis J., Steward L.E. (2008). Novel chimeras of botulinum neurotoxins a and e unveil contributions from the binding, translocation, and protease domains to their functional characteristics. J. Biol. Chem..

[286] Strotmeier J., Mahrhold S., Krez N., Janzen C., Lou J., Marks J.D., Binz T., Rummel A. (2014). Identification of the synaptic vesicle glycoprotein 2 receptor binding site in botulinum neurotoxin A. FEBS Lett..

[287] Rummel A., Mahrhold S., Bigalke H., Binz T. (2011). Exchange of the HCC domain mediating double receptor recognition improves the pharmacodynamic properties of botulinum neurotoxin. FEBS J..

[288] Band P.A., Blais S., Neubert T.A., Cardozo T.J., Ichtchenko K. (2010). Recombinant derivatives of botulinum neurotoxin A engineered for trafficking studies and neuronal delivery. Protein Expr. Purif..

[289] Vessely C., Estey T., Randolph T.W., Henderson I., Cooper J., Nayar R., Braun L.J., Carpenter J.F. (2009). Stability of a trivalent recombinant protein vaccine formulation against botulinum neurotoxin during storage in aqueous solution. J. Pharm. Sci..

[290] Vazquez-Cintron E.J., Vakulenko M., Band P.A., Stanker L.H., Johnson E.A., Ichtchenko K. (2014). Atoxic derivative of botulinum neurotoxin A as a prototype molecular vehicle for targeted delivery to the neuronal cytoplasm. PLoS ONE.

[291] Pier C.L., Tepp W.H., Bradshaw M., Johnson E.A., Barbieri J.T., Baldwin M.R. (2008). Recombinant holotoxoid vaccine against botulism. Infect. Immun..

[292] Bradshaw M., Tepp W.H., Whitemarsh R.C., Pellett S., Johnson E.A. (2014). Holotoxin Activity of Botulinum Neurotoxin Subtype A4 Originating from a Nontoxigenic *Clostridium botulinum* Expression System. Appl. Environ. Microbiol..

[293] Chen S., Barbieri J.T. (2009). Engineering botulinum neurotoxin to extend therapeutic intervention. Proc. Natl. Acad. Sci. USA.

[294] Simpson L. (2013). The life history of a botulinum toxin molecule. Toxicon.

[295] Montecucco C., Rasotto M.B. (2015). On botulinum neurotoxin variability. mBio.

[296] Lindstrom M., Myllykoski J., Sivela S., Korkeala H. (2010). Clostridium botulinum in cattle and dairy products. Crit. Rev. Food Sci. Nutr..

[297] Kruger M., Grosse-Herrenthey A., Schrodl W., Gerlach A., Rodloff A. (2012). Visceral botulism at dairy farms in schleswig holstein, germany: Prevalence of clostridium botulinum in feces of cows, in animal feeds, in feces of the farmers, and in house dust. Anaerobe.

[298] Galey F.D. (2001). Botulism in the horse. Equine Pract..

[299] Elad D., Yas-Natan E., Aroch I., Shamir M.H., Kleinbart S., Hadash D., Chaffer M., Greenberg K., Shlosberg A. (2004). Natural clostridium botulinum type C toxicosis in a group of cats. J. Clin. Microbiol..

[300] Borst G.H., Lambers G.M., Haagsma J. (1986). Type-C botulism in dogs. Tijdschr. Diergeneeskd..

[301] Whitemarsh R.C., Strathman M.J., Chase L.G., Stankewicz C., Tepp W.H., Johnson E.A., Pellett S. (2012). Novel application of human neurons derived from induced pluripotent stem cells for highly sensitive botulinum neurotoxin detection. Toxicol. Sci..

[302] Raptis A., Torrejon-Escribano B., Gomez de Aranda I., Blasi J. (2005). Distribution of synaptobrevin/VAMP 1 and 2 in rat brain. J. Chem. Neuroanat..

[303] Karalewitz A.P., Fu Z., Baldwin M.R., Kim J.J., Barbieri J.T. (2012). Botulinum neurotoxin serotype C associates with dual ganglioside receptors to facilitate cell entry. J. Biol. Chem..

[304] Blasi J., Chapman E.R., Yamasaki S., Binz T., Niemann H., Jahn R. (1993). Botulinum neurotoxin C1 blocks neurotransmitter release by means of cleaving HPC-1/syntaxin. EMBO J..

[305] Blasi J., Chapman E.R., Link E., Binz T., Yamasaki S., de Camilli P., Sudhof T.C., Niemann H., Jahn R. (1993). Botulinum neurotoxin A selectively cleaves the synaptic protein SNAP-25. Nature.

[306] Peng L., Liu H., Ruan H., Tepp W.H., Stoothoff W.H., Brown R.H., Johnson E.A., Yao W.D., Zhang S.C., Dong M. (2013). Cytotoxicity of botulinum neurotoxins reveals a direct role of syntaxin 1 and SNAP-25 in neuron survival. Nat. Commun..

[307] Wang D., Zhang Z., Dong M., Sun S., Chapman E.R., Jackson M.B. (2011). Syntaxin requirement for Ca^2+^-triggered exocytosis in neurons and endocrine cells demonstrated with an engineered neurotoxin. Biochemistry.

[308] Wiley R.G., Kline R.H.t., Vierck C.J. (2007). Anti-nociceptive effects of selectively destroying substance P receptor-expressing dorsal horn neurons using [Sar9,Met(O2)11]-substance P-saporin: Behavioral and anatomical analyses. Neuroscience.

[309] Wiley R.G., Lappi D.A. (2003). Targeted toxins in pain. Adv. Drug Deliv. Rev..

[310] Wiley R.G., Lappi D.A. (1997). Destruction of neurokinin-1 receptor expressing cells *in vitro* and *in vivo* using substance p-saporin in rats. Neurosci. Lett..

[311] Wiese A.J., Rathbun M., Butt M.T., Malkmus S.A., Richter P.J., Osborn K.G., Xu Q., Veesart S.L., Steinauer J.J., Higgins D. (2013). Intrathecal substance p-saporin in the dog: Distribution, safety, and spinal neurokinin-1 receptor ablation. Anesthesiology.

[312] Mantyh P.W., Rogers S.D., Honore P., Allen B.J., Ghilardi J.R., Li J., Daughters R.S., Lappi D.A., Wiley R.G., Simone D.A. (1997). Inhibition of hyperalgesia by ablation of lamina I spinal neurons expressing the substance p receptor. Science.

[313] Choi J.I., Koehrn F.J., Sorkin L.S. (2012). Carrageenan induced phosphorylation of akt is dependent on neurokinin-1 expressing neurons in the superficial dorsal horn. Mol. Pain.

[314] Mustafa G., Anderson E.M., Bokrand-Donatelli Y., Neubert J.K., Caudle R.M. (2013). Anti-nociceptive effect of a conjugate of substance p and light chain of botulinum neurotoxin type A. Pain.

[315] Keith F., John C. (2010). Targeted secretion inhibitors-innovative protein therapeutics. Toxins.

[316] Edwards J.G. (2014). TRPV1 in the central nervous system: Synaptic plasticity, function, and pharmacological implications. Prog. Drug Res..

[317] Duggan M.J., Quinn C.P., Chaddock J.A., Purkiss J.R., Alexander F.C., Doward S., Fooks S.J., Friis L.M., Hall Y.H., Kirby E.R. (2002). Inhibition of release of neurotransmitters from rat dorsal root ganglia by a novel conjugate of a clostridium botulinum toxin a endopeptidase fragment and erythrina cristagalli lectin. J. Biol. Chem..

[318] Apland J.P., Adler M., Oyler G.A. (2003). Inhibition of neurotransmitter release by peptides that mimic the n-terminal domain of SNAP-25. J. Protein Chem..

[319] Park T.Y., Shin M.J., Park S.D., Lee S.K. (2013). Alleviation of abnormal synaptic neurotransmitter release by cell-permeable form of the truncated SNAP-25 upon transcutaneous delivery. Neurosci. Lett..

[320] Burke G.S. (1919). The occurrence of bacillus botulinus in nature. J. Bacteriol..

[321] Rossetto O., Pirazzini M., Montecucco C. (2014). Botulinum neurotoxins: Genetic, structural and mechanistic insights. Nat. Rev. Microbiol..

